# Co-production using Theory of Change in the development of the SUCCEED Africa community-based rehabilitation intervention for people living with psychosis in West and Southeast Africa

**DOI:** 10.1371/journal.pmen.0000668

**Published:** 2026-09-11

**Authors:** Julian Eaton, Olubukola Omobowale, Rita Tamambang, Rachel Greenley, Lloyd Dzapasi, Musa Buyanga, Martin Nkhata, Olayinka Bamidele, Anthony Sefasi, Olayinka Omigbodun, Rebecca Esliker, Olayinka Aturu, Adeola Oduguwa, Olusegun Ogunmola, Alhaji Koroma, Epiphania Munetsi, Tom Shakespeare, Grace K. Ryan

**Affiliations:** 1 Department of International Public Health, Liverpool School of Tropical Medicine, Liverpool, United Kingdom; 2 Department of Community Medicine, College of Medicine, University of Ibadan, Ibadan, Nigeria; 3 Department of Psychiatry, College of Medicine, University of Ibadan, Ibadan, Nigeria; 4 Centre for Global Mental Health, London School of Hygiene and Tropical Medicine, London, United Kingdom; 5 Faculty of Medicine and Health Sciences, University of Zimbabwe, Harare, Zimbabwe; 6 Department of Mental Health, School of Nursing, Kamuzu University of Health Sciences, Blantyre, Malawi; 7 Department for Clinical Counselling, University of Makeni, Makeni, Sierra Leone; University of Canterbury, NEW ZEALAND

## Abstract

Psychosis and other severe mental health conditions cause substantial disability and reduced functioning across a number of domains. Services in low- and middle-income settings are often weak, and predominantly biomedical. Community-Based Rehabilitation (CBR) offers a comprehensive and person-centred approach that is aligned to effective case management models. The Support, Comprehensive Care and Empowerment for People with Psychosocial Disabilities (SUCCEED) programme was co-produced with people living with psychosis in four sub-Saharan countries, with a view to improving quality of life, mental health outcomes, and community participation. This paper described the intervention development process, using multiple iterations of Theory of Change (ToC), incorporating situation analysis, formative research and piloting. We present these iterations as results, using the GUIDED framework for intervention development, and the intervention is described using the TIDieR framework. We conclude that ToC can promote participatory processes of intervention co-development, as well as generate research questions. Interventions must remain flexible, and be open to revision, even if having core active ingredients.

## Introduction

### Severe mental health conditions in low-resource settings

People living with severe mental health conditions often have complex needs across different domains - such as interpersonal relationships, housing, law, and livelihoods - as well as health services [1]. Lack of access to services, social isolation, and physical neglect contribute to a 15–20 year reduction in life expectancy among people with schizophrenia [2]. Human rights abuses are common, and poor quality of life is often reported [3].

In very low-resourced settings, like many communities in sub-Saharan Africa, services are typically hard to access, and statutory social safety nets are extremely limited. Families, informal caregivers and wider communities provide the mainstay of care. When these support networks break down, people with severe mental health conditions often only have specialist institutions as an option, which are sometimes geographically or financially inaccessible. In the absence of accessible, appropriate care pathways, these systemic gaps can leave individuals without adequate support, exposing them to informal or traditional treatments that may provide little more than containment, or risk prison or street homelessness [4].

### Comprehensive care for people with severe mental health conditions

The need to provide accessible, comprehensive support that goes beyond health care is well recognised, and there is a long history of innovation originating from diverse settings, including from the African continent. In Nigeria, Thomas Adeoye Lambo established a village system in the 1950s, where people attending the hospital in Aro, Abeokuta, stayed in local villages to maintain social skills while receiving treatment [5]. Similar community-oriented models have since emerged in several African and Global South settings. Older examples include the centuries-old community-based social support in Geel, Belgium [6].

More recently, case management - facilitating access to services across different sectors through the support of a key worker - has been shown to be effective in reducing relapse and hospitalisation, and improving functioning [7]. A key worker, or case manager, is a single designated worker who takes responsibility for coordinating a service user’s care and support across health, social and livelihood sectors [8]. This relies on signposting service users to a range of available services using a person-centred approach that tailors support based on individual needs. Most case management literature is drawn from high-income and non-African settings, but more recent work from sub-Saharan Africa, like the RISE trial of community-based rehabilitation for schizophrenia in Ethiopia [9], is beginning to build a more contextually relevant evidence base.

Assertive Community Treatment [10] is perhaps the best known of these approaches, though similar models have emerged, which all involve a case manager providing intensive support to a small number of clients [11]. Other examples of comprehensive care, some of which have emerged from the Global South, include intensive support for homeless people with severe mental conditions in therapeutic communities [12], and self-help groups which, in addition to socio-emotional support between peers, may focus on livelihoods, stigma reduction or more explicit rights-based advocacy [13].

When considering the comprehensive needs of people with severe mental health conditions like psychoses, it is helpful to draw on disability research, which emphasises not only the impairment associated with a health condition, but also the social and environmental factors that influence a person’s ability to meaningfully participate in society [14]. This social model of disability is the basis of Community-Based Rehabilitation (CBR), which has been applied to psychosocial disabilities, including in Africa [9,15]. In contrast to the case management typically used in high-income countries, where supporting access to mainstream statutory services is often a central aim, CBR looks to also maximise the support accessed from care providers in communities - something that is extremely important where formal systems are weak [16]. In this paper, care providers in the community refers to the range of formal and informal actors who provide some form of care or support to people with mental health conditions in everyday settings, including family and household caregivers, community and religious leaders, traditional and faith healers, community health workers, peer supporters, and civil society organisations.

### SUCCEED Africa

In response to these needs and service gaps, we co-produced the design and evaluation of a comprehensive, community-based intervention to support people with psychoses in four countries in sub-Saharan Africa: Malawi, Nigeria, Sierra Leone and Zimbabwe. Support, Comprehensive Care and Empowerment for People with Psychosocial Disabilities (SUCCEED) is a six-year programme, funded by UK Aid to generate evidence on comprehensive approaches to support for people with severe mental conditions in sub-Saharan Africa. This paper describes the participatory process through which the SUCCEED intervention was developed for subsequent evaluation. The objectives of this paper are: (i) to describe the iterative, co-produced Theory of Change (ToC) process used to develop the SUCCEED CBR intervention for people living with psychosis across four sub-Saharan African settings; (ii) to present the evolving ToC maps (site-specific, cross-site pre-pilot, and cross-site post-pilot) and the intervention components derived from them; (iii) to describe the final intervention using standardised reporting frameworks (GUIDED and TIDieR); and (iv) to reflect on the methodological strengths, limitations, enablers and barriers of this participatory development approach.

## Methods

Following evidence-based recommendations to improve transparency of the intervention development process, we describe the methods used for SUCCEED’s intervention development below using the GUIDED framework for reporting [17]. The resulting intervention is described using the TIDieR framework for intervention description and replication [18].

### Setting

The SUCCEED programme was established in 2019, with a set of clear principles and values drawn from team development work across five sites (Blantyre in Malawi, Ibadan in Nigeria, Makeni in Sierra Leone, Harare in Zimbabwe, and the coordinating centre in London, UK). Across the four implementation settings there are important contextual similarities and differences that shape both the development and potential replicability of the intervention. Similarities include weak formal mental health services with limited specialist coverage, high levels of community stigma, active traditional and faith-based pathways to care, and limited statutory social protection. Key differences include the urban-rural composition of each site, the economic structures that shape livelihoods options, the relative maturity and reach of civil society organisations, linguistic and cultural diversity, and specific country histories, notably the post-conflict context in Sierra Leone. These similarities and differences are described in more detail in a four-country cross-site situation analysis [19], and are reflected in the decision to build the intervention around a common core with explicit provision for local adaptation.

### Target population

In order to provide clarity, the target group was defined as adults with lived experience of psychosis - including conditions like schizophrenia and bipolar affective disorder. This group is recognised as being particularly socially excluded, with little access to services that promote sustained wellbeing in the community. While there were methodological reasons for a tight sample definition, we also felt that it was important to have ‘caseness’ defined by non-specialists, so the means of identifying and screening potential service users was deliberately made feasible outside of the context of specialist services. A prior formal clinical diagnosis is not a precondition for engagement with the SUCCEED intervention. Eligibility is established through community-level identification of presentations consistent with psychosis, including schizophrenia-spectrum and bipolar affective disorder, using the Community Informed Detection Tool, followed by a structured screening process operable by non-specialists. Where specialist clinical confirmation is accessible, it is sought to inform ongoing care, but it is not required for entry to the intervention. This choice reflects a deliberate design commitment to identification of need in settings where formal diagnostic services are often inaccessible.

### Approach to intervention development

SUCCEED Africa uses Theory of Change (ToC) to guide intervention development and evaluation. ToC has been used extensively in global mental health as an approach that enables collaborative intervention development and testing of postulated pathways to change [20]. Initially, a high level framework was developed, as a means of establishing the basic premise of the work, and designing the research. This was necessarily developed by a small group, made up of the Principal Investigators across the sites, and a number of other researchers and people with lived experience. Once the research started, we used ToC as a means of using evidence and experience-informed considerations to develop a hypothetical pathway to a desired change. This was an iterative process, incorporating evidence and experience as the programme progressed, informing appropriateness of postulated mechanisms that might lead to specified outcomes on a pathway to a final defined impact. ToC was the overarching framework that defined both the intervention design, and the research methods used to test the assumptions made in the intervention model that arose from it.

## Stakeholder involvement and guiding principles

### Co-production

The first principle that underpinned the programme included recognising the central importance of learning from people living with the mental health conditions, alongside and complementing established psychiatric and global mental health traditions in scientific literature. Our understanding of co-production builds on Epstein’s analysis of the coproduction of knowledge between affected communities and scientific communities [21], and on more recent conceptualisations in global mental health that frame co-production as a continuum of meaningful involvement across the research cycle, as described in Jakobsson and colleagues’ scoping review of co-produced psychosis research in Africa and elsewhere [22].

In keeping with this, the Programme sought to apply best practice in co-production, ensuring that knowledge is generated through meaningful involvement of people affected [21]. In practice this was achieved through a number of mechanisms, for example by employing peer researchers in each site (at least two people), who participated in all aspects of the research. Similarly, the unique role of peer support as a therapeutic component in the intervention was incorporated, emerging from the principle of co-production, and learning from projects like Brain Gain [23] and UPSIDES [24], which both demonstrate outcomes of value to our stakeholders. Throughout this paper we use co-production as the overarching term for the participatory approach to intervention development taken by SUCCEED. We use the term co-deign to refer specifically to the structured workshop sessions in which teams jointly designed intervention components.

A governance structure was also established that facilitated input from a Lived Experience Advisory Panel (LEAP), independently reviewing and advising programme direction at the international level, and membership of organisations of people with psychosocial disabilities in Local Advisory Groups in each country. These LAGs also provided an opportunity for local stakeholders to feed into the research and provided a level of accountability to the communities where research was located.

### Co-production processes and stakeholder engagement

Stakeholders engaged in the co-production process included people with lived experience of psychosis (as peer researchers employed in each site, peer support workers during the pilot, and members of the international Lived Experience Advisory Panel and country-level Local Advisory Groups), family caregivers, mental health professionals across primary, secondary and specialist levels, community support workers and supervisors, community and religious leaders, traditional and faith healers, staff of partner civil society organisations, researchers, and policy and service delivery actors. Participants were identified through the existing relationships of site research teams and partner civil society organisations, with targeted outreach to ensure that each workshop included people with lived experience of psychosis and family caregivers alongside professional and academic contributors. The international Lived Experience Advisory Panel was constituted through open expression of interest coordinated with lived experience organisations across the four countries. Local Advisory Groups included representatives of organisations of people with psychosocial disabilities, caregiver associations, civil society, and local service providers.

To address power dynamics in workshops, several practical measures were taken. Peer researchers co-facilitated sessions alongside academic facilitators, and separate lived-experience-only spaces were offered at key points for reflection and preparation. Workshops were structured so that contributions from academic and clinical participants were explicitly followed by responses from people with lived experience and caregivers, rather than the reverse. Facilitators actively named and managed disparities in status, professional language and familiarity with research processes. Materials were simplified and offered in relevant local languages, with translation where needed. Workshop schedules included breaks and informal time to enable relationship-building, and honoraria and travel reimbursement were provided for participants with lived experience. The international LEAP reviewed each major version of the ToC and the overall direction of the programme.

### Social model of disability

We drew on broad research traditions, incorporating disability as well as public health and global mental health, in addition to seeking out research carried out in contexts most relevant to our work. Drawing on the United Nations Convention on the Rights of Persons with Disabilities (UNCRPD) and on foundational work in disability studies including Mulvany’s analysis of its application to mental health [14], this tradition shifts the focus from impairment alone to the social and structural factors that shape participation.

**Fig 1:**
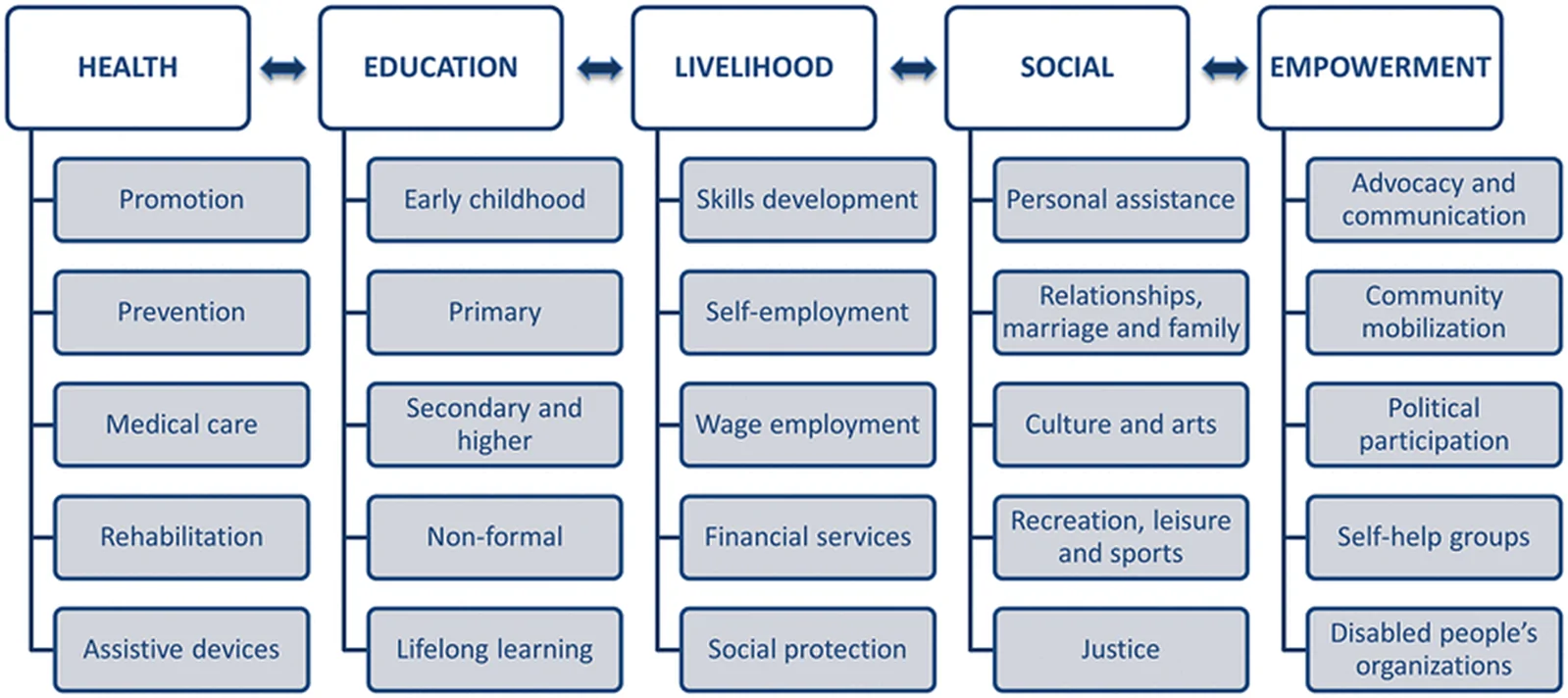
CBR Framework used as a basis for the intervention (from WHO CBR Guidelines) [25].

The basis of access to any services is inalienable human rights, to which states and other duty bearers must enable access. CBR research comes from this tradition, though inevitably needs to grapple with the challenges of enabling access to rights in resource-constrained environments. Published by the WHO in 2010, the CBR Guidelines includes a section on mental health [25]. They cover health, education, social, empowerment and livelihood domains, each of which has a sub-level list of main areas of activity (see Fig 1). CBR was developed in and for contexts similar to those within which we planned to work, and was the high-level framing that was used for the intervention.

### Sustainability and scalability

Finally, we were also committed to culturally appropriate, feasible, and sustainable interventions, with the ambition of maximising potential impact with scaled adoption beyond the research carried out. Alongside the outcome evaluation in the full randomised controlled trial, we include a process evaluation, which takes a realist approach, testing assumptions in our ToC by examining the mechanisms through which interventions produce particular outcomes in particular contexts, using mixed methods [26]. Practical considerations of these principles included not making the delivery of the intervention too expensive (recognising that research funds are unlikely to be available in future implementation), using only the human resources available in these contexts, and remaining committed to the principle of mobilising existing community resources where possible.

### Project timeline

The intervention development work described in this paper spanned the period 2019–2024, proceeding in five overlapping phases: (i) programme establishment and development of guiding principles across the five sites (2019); (ii) situation analyses, formative research and systematic reviews, and site-specific ToC (Stage 1) maps, conducted largely virtually during COVID-19 restrictions (2020–2021); (iii) cross-site ToC (Stage 2) development, intervention manualisation and training material development (2022); (iv) pilot training and four-month pilot implementation across all four sites (2023); and (v) ToC (Stage 3) revision during an in-person cross-site meeting in Blantyre, Malawi (early 2024), leading into the full Randomised Controlled Trial and process evaluation phase (2024 onward). A detailed timeline is provided in S1 Table.

## Development process

### Situation analysis and formative research

The first two years of the SUCCEED programme were dedicated to understanding the contexts in which people lived and in what ways this influenced their experience of living with a severe mental health condition. It was as part of this process that the country-specific ToCs were developed (ToC stage1).

A comprehensive situation analysis using a framework adapted from WHO QualityRights [27] and the PRIME [28] project drew on published literature and other data sources. WHO QualityRights provides a structured, rights-based lens for assessing mental health services and identifying gaps against internationally recognised human rights standards, which was directly relevant given our explicit commitment to the social model of disability and the UNCRPD. PRIME was selected because it offers a tested template for conducting situation analyses in low- and middle-income mental health system contexts. This included reviews of policy, service infrastructure, financing and local data systems, though there were many gaps in information. Findings were compared and published in a cross site analysis [19], providing an outline of the available resources, and barriers to equal participation, for people with psychosocial disabilities.

Finally, systematic reviews were carried out, examining provision of care for people with psychosis in Africa [29], and effectiveness and appropriateness of CBR as a service model [16].

This phase also included defining priority outcomes and how they would be measured, alongside gaining insights into what an effective and culturally appropriate intervention might contain in these contexts. People with lived experience were key to these processes, informing the intervention and research (developing research questions and interview topic guides), analysis and interpretation.

### Operationalising the intervention

An initial series of online training workshops conducted to introduce the concept of Theory of Change to country team members, then focal persons from each country facilitated co-produced ToC sessions in their country (ToC stage 1). A series of online workshops were held, where the initial country-specific ToCs was reviewed, and the formative research above was incorporated into a cross-site ToC (stage 2), leading to a narrative description of different elements of the proposed intervention. Consideration was made of feasibility and acceptability in the contexts we would be delivering the intervention. After an initial inability to meet in person due to COVID-19, annual cross-site meetings were held in Nigeria and Malawi, where the draft interventions were further refined on the basis of the ToC. Outputs from co-production workshops (facilitator notes, flipchart sheets, photographs of ToC maps, and summary reports) were compiled by site focal persons and shared across the programme. A core writing group, including representatives from each country and peer researchers, synthesised these outputs into successive drafts of the cross-site ToC by mapping shared outcomes, assumptions and mechanisms across the four sites and identifying where sites diverged. Each draft was circulated to all sites for feedback, and divergences were resolved through structured virtual meetings and, where feasible, face-to-face cross-site meetings, including annual in-person meetings in Ibadan, Nigeria and Blantyre, Malawi. Decisions were made through a consensus-seeking approach. Where consensus could not be reached on a specific activity or outcome, the default was to preserve site-level flexibility, rather than impose a uniform position. The Lived Experience Advisory Panel was consulted on each major version of the ToC.

The intervention was subsequently manualised and used to develop comprehensive training materials (discussed below) to prepare the delivery teams for evaluation in a pilot study. These materials were adapted across sites but retained core content to ensure fidelity to the ToC and comparability across country contexts.

### Ethics and safeguarding

The SUCCEED programme, including the co-production activities described in this paper, received ethical approval from the London School of Hygiene and Tropical Medicine Research Ethics Committee (Ref: 28346) and from the ethics committees in each country: the University of Ibadan and University College Hospital Ethics Committee for Nigeria (UI/EC/224/0214); the Kamuzu University of Health Sciences Research Ethics Committee for Malawi (P.03/23/4032); the Sierra Leone Ethics and Scientific Review Committee (SLESRC: 008/02/2024); and the Medical Research Council of Zimbabwe (MRCZ/A/3155). Informed consent was obtained from all workshop and research participants, with consent processes adapted for literacy level and delivered in relevant local languages. Specific protections for participants with lived experience of psychosis included pre-workshop orientation, availability of a trusted support person, scheduled breaks and check-ins, a designated site team member responsible for distress response, and defined referral pathways to existing mental health services. Capacity to consent was assessed at each point of engagement, and participants were free to decline involvement or withdraw at any stage without any consequence to access to services. Safeguarding procedures, including a designated safeguarding lead in each country, a documented escalation and referral pathway, and peer support structures for PSWs, are described in the Results section.

### Piloting

The pilot study for SUCCEED provided an opportunity to systematically test the content of the intervention in a real-world setting, with detailed process evaluation of how its components worked [30]. Following development of the training materials based on the intervention, all implementation personnel were provided with a 2-week training programme covering background information about mental health, with specific guidance on intervention components, and sessions on assessment of needs, reporting, self-care etc. Training was assessed using the ENACT tool to evaluate competency in the delivery of psychosocial interventions [31]. These assessments informed revisions such as more role play, and peer-led supervision. Following the pilot, core components of the intervention remained intact, but more systematic documentation, and structured community engagement were added. The final revision prior to the full evaluation in an RCT (ToC stage 3) used the pilot results to update the stage 2 ToC during a face to face cross-site yearly meeting in Blantyre, Malawi. With representatives of all countries present, outcomes, interventions, and assumptions within the TOC were revised, which led to updates of the intervention and training.

## Results

These results are presented to address components of the GUIDED checklist. See S2 Table.

### ToC stage 1: Site-specific theory of change maps

**Fig 2:**
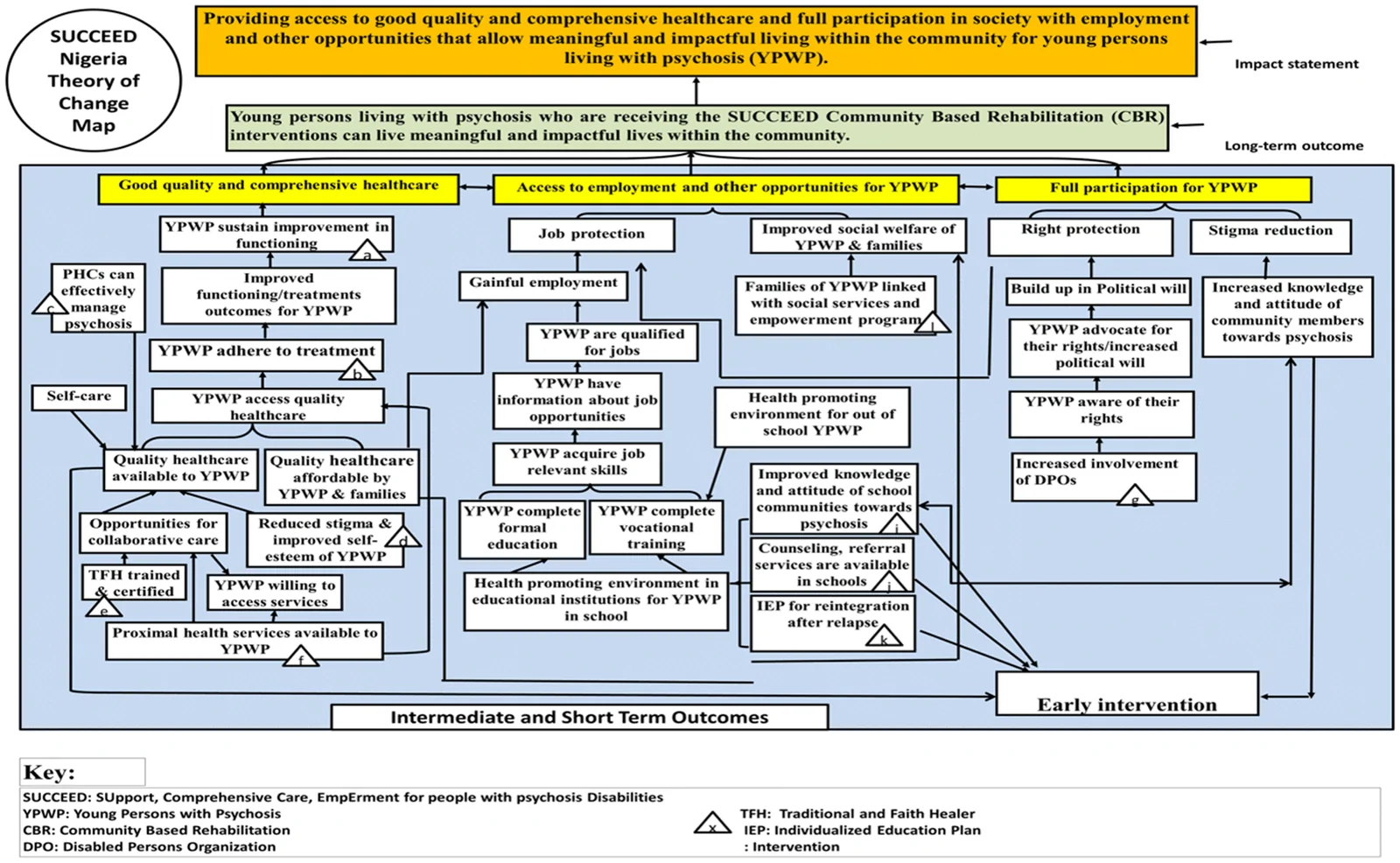
The SUCCEED Nigeria Team in-country Theory of Change Map.

The initial ToCs were unique to each country, but were all based around the five domains of CBR. The four teams considered the different factors that could lead logically through intermediate outcomes to a final intended impact were documented. (see Fig 2 for the Nigeria ToC as an example). In all the country-specific ToCs, secure income, access to health care, social participation and addressing exclusion were emphasised.

### ToC stage 2: Cross-site theory of change map (pre-pilot)

**Fig 3:**
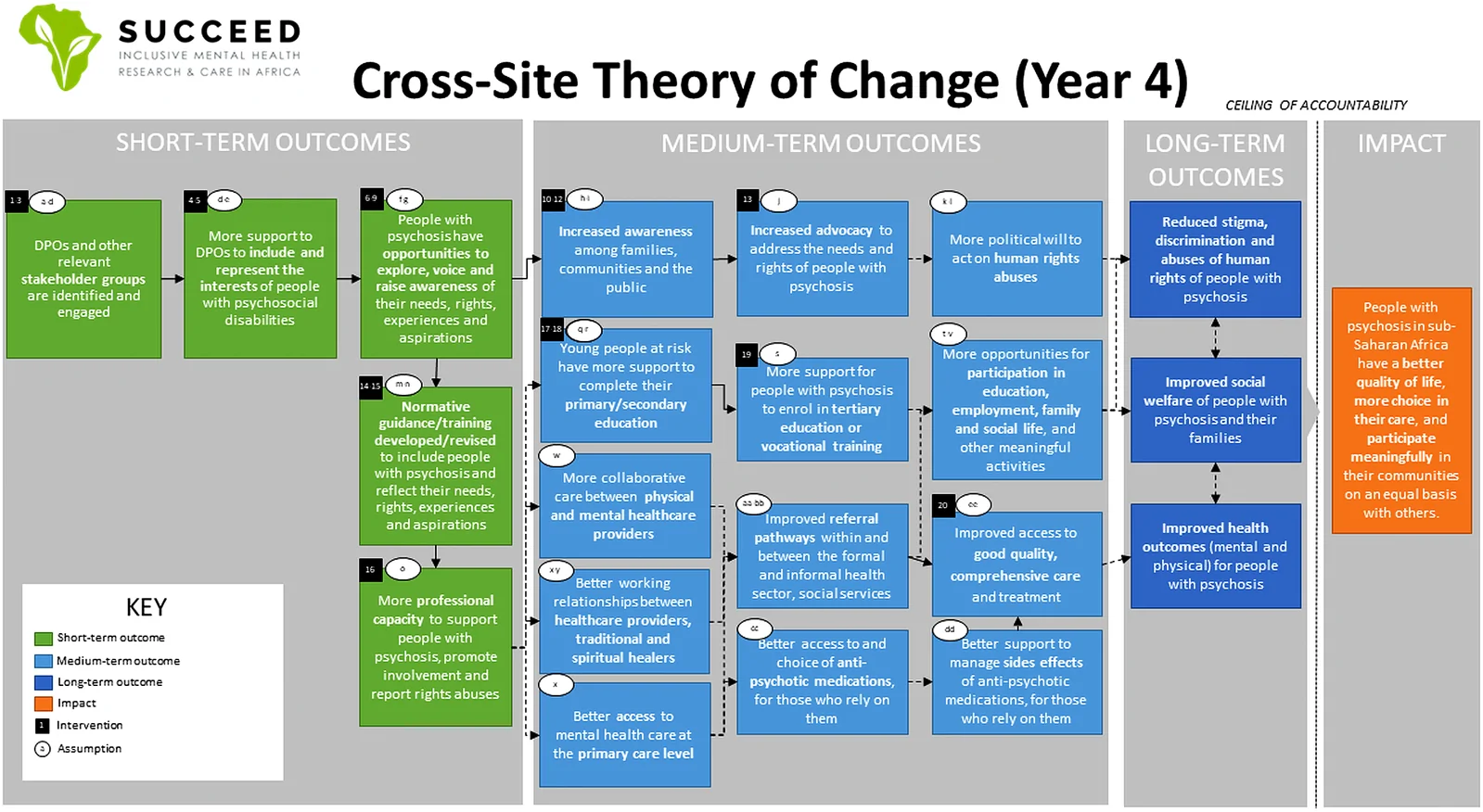
The SUCCEED common cross-site Theory of Change Map prior to piloting.

The common cross-site Theory of Change map was developed through a series of virtual meetings involving teams from all participating countries, and utilising the four in-country ToC maps they had created. See Fig 3. The different long-term outcomes identified by countries were synthesized into a central theme focused on *enhancing quality of life, increasing choice of care, and enabling meaningful participation in community life*. Besides detailing the intervention components, the cross-site ToC map also included a compilation of assumptions and outcomes—organized into short-term, mid-term, and long-term categories necessary to achieve the ultimate outcome. These contributed to research questions.

### Intervention components

Four focus areas of activities arose from gaps and proposed solutions identified in the cross-site ToC: i) provision of case management and access to community resources to address weak formal services, ii) peer support to promote wellbeing, iii) livelihood support to address pernicious poverty, and (iv) community engagement through CMHFs emerged after stigma was identified as a major factor reducing quality of life. These became the core ingredients of the intervention, which were further refined during design workshops (see Table 1). Their implementation was facilitated by community engagement, training, supervision and monitoring of delivery.

### Intervention delivery

SUCCEED’s case management approach is delivered by dyads made up of one Community Support Worker (CSW) and one Peer Support Worker (PSW), supporting approximately ten service users in a defined geographic area. CSWs are adults with at least secondary-level education, resident in the programme catchment area, and with prior experience of community work. PSWs are adults with lived experience of psychosis who are stable and in recovery, resident in the programme area, and willing to disclose their lived experience in a work context. Each country team is supported by one Supervisor and one Peer Supervisor. All personnel complete a two-week structured training programme covering mental health literacy, each intervention component, needs assessment, safeguarding, and self-care, followed by field-based mentorship during initial home visits, peer group sessions and Community Mental Health Forums. The Support Workers deliver at least one in-person home visit and one telephone review per service user per month, alongside regular peer group sessions and an annual Community Mental Health Forum. Supervision takes the form of monthly team meetings and ongoing field accompaniment by Supervisors. Full details of intervention components, providers, dose and fidelity are provided in the TIDieR description (S1 Text).

A structured process of identification, engagement, screening and assessment of needs was defined in detail. For example the Community Informed Detection Tool developed in Nepal [32], was adapted for the SUCCEED sites. This was facilitated by its use as part of Community Mental Health Forums, held annually in each community to engage with communities, help identify cases for assessment, and promote community support for people with mental conditions [33]. On identification, engagement with service users and their caregivers occurs, followed by a detailed needs assessment, again adapted based on previous work with similar target groups [34]. The assessment of needs was used as a basis for establishing a personalised care plan organised across CBR domains, but primarily defined by the preferences of the service user.

**Table 1:** Description of intervention components.

Intervention Component	Description
**Establishing the service**	- Assess needs and available resources in the community - Establish appropriate governance and local ownership, including participation of people with lived experience - Adapt the intervention to local needs, local resources, language, culture - Recruit and train staff (using SUCCEED job descriptions and training materials)
**Community Mental Health Forums (CMHFs) [33]**	Annual community-based meetings where relevant community stakeholders are invited to an open meeting. Aims are to: - listen and understand local needs, resources, beliefs about mental health - build trusting relationships with local community stakeholders - address stigmatising beliefs and attitudes. Contact intervention with people with lived experience is crucial
**Identification and recruitment**	Appropriate clients to receive the intervention are identified through three routes: - Referral from existing health services - Active case finding through the CMHFs and community engagement - Use Community Informed Detection Tool (CIDT) [35] and screening with inclusion criteria
**Case Management**	- Link service user with Community Support Worker and Peer Support Worker to oversee care and support across different sectors - Establish regular review meetings (monthly in-person and by telephone) with CSW and PSW - Support access to available community and formal services, including health, education/livelihood, and social participation - May involve accompaniment (initially), and institutional readiness visits to improve service user reception
**Assess specific needs and available resources**	- Conduct individual and family needs assessment using Needs Assessment tool - Set out personal plans based on person-centred recovery approach
**Peer Support**	- Establish (or make use of existing) groups that meet regularly (e.g., monthly) in a convenient location - Includes time for service users alone, but also with family members
**Livelihood support**	- Identify realistic aspirations of service users based on their needs assessment. Options are usually based on individual career or education aim, or group-based resource mobilisation activity - Use Asset Based Community Development (ABCD) [36] approach for group activities, and Individual Placement and Support (IPS) [37] for individual cases
**Supervision and quality assurance**	Provide regular supervision and support of PSWs/CSWs - One Supervisor for 10 teams routinely accompanies to observe activities to ensure quality and good practice - One Peer Supervisor supports PSWs, and if necessary, temporarily cover for work of PSW - Collect and report on routine programme data using SUCCEED M&E forms - Meet monthly as a team for personal and skills development and support

### ToC stage 3: Cross-site theory of change map (post-pilot)

#### Piloting the intervention.

The pilot evaluation involved establishment of a single dyad of providers - one CSW and one PSW - in each of the four SUCCEED country sites. Recruitment of CSWs and PSWs was led by the partner civil society organisation in each country (ASIDO Foundation in Nigeria, the Mental Health Users and Carers Association, MeHUCA, in Malawi, the Mental Health Coalition in Sierra Leone, and the Zimbabwe National Association for Mental Health, ZIMNAMH), rather than directly by the academic research team, in order to model the recruitment pathway envisaged for any future scale-up beyond a research context. All staff signed commitments to the SUCCEED safeguarding and confidentiality standards following training.

Following recruitment, training was provided, and the intervention implemented and evaluated. These local civil society organisations were chosen as typical of those envisaged for any future scale-up. This arrangement enabled the evaluation of real-world feasibility and acceptability, and we were disciplined about keeping within typical resource limitations.

The pilot lasted for four months, to allow observation of the intervention’s key components, including home visits, group-based livelihoods sessions, and informal peer support. We recognised that more sustained delivery would likely be required to observe meaningful changes in outcomes such as health status or livelihood outcomes.

The pilot generated a number of insights into the practicalities of implementation, training and supervision needs, and the operational dynamics of lay-delivered mental health support in low-resource, community-based settings. These were documented as updated activities and assumptions (see S3 Table), and a number of changes were made to the ToC and intervention:

1. The structural conditions within which peer support workers operated, including role ambiguity, limited locally-relevant training and supervision infrastructure, financial precarity, and broader systemic pressures within under-resourced mental health services, created challenging working conditions that at times made it difficult for PSWs to sustain their roles. In response, PSWs were given additional flexibility in their work, including the ability to take time off without losing income or job security; one or two floating PSWs were recruited per site to ensure continuity of service; a Peer Supervisor role was created in each site to provide dedicated peer-led supervision and advocacy; and additional locally-relevant training, supervision and team-based support were introduced
2. Safeguarding was substantially strengthened as a result of the pilot. Safeguarding was incorporated into the two-week induction training for all personnel, covering identification and response to risk of harm to self, risk of harm from others, child safeguarding, and staff wellbeing. Dedicated peer-led supervision structures were established for PSWs, recognising the emotional labour of the role and the potential for distress. Safeguarding review was introduced as a standing item in monthly team meetings, and any safeguarding incidents or near-misses were logged and reviewed across sites to inform iterative revision of procedures.
3. The Intervention Manual did not describe some elements of the intervention in sufficient detail, so that, even after training, implementation was varied. Post-pilot discussions highlighted these inconsistencies, and led to collectively agreed activities, which were manualised and included in updated training. Examples included the frequency of home visits, and participation of caregivers in livelihood activities. It was recognised that some flexibility was needed, based on local contexts, for example dealing with different levels of availability of medication.
4. Livelihoods activities had the greatest diversity in implementation across countries, based on differing economic contexts, as well as preferences and experience of different sites. It was decided that while needs assessment and options available for group or individual level support should be consistent, flexibility was needed to meet participants’ preferences.
5. The intensity of routine monitoring of the programme was excessive, with Supervisors spending more time doing this than providing practical support. The intensity of monitoring was subsequently reduced, and emphasis placed on the support that Supervisors provide.
6. In addition to the 2 weeks of training, it was clear that the best learning for CSWs and PSWs occurred once they started work, so a more formal field-based learning element was introduced, with Supervisors or trainers accompanying, the first Community Mental Health Forum, group session, or home visit.
7. Due to experience of some staff dropping out of the work soon after training, all sites trained more CSWs and PSWs than would be needed, so that trained staff were available to recruit if needed.

**Fig 4:**
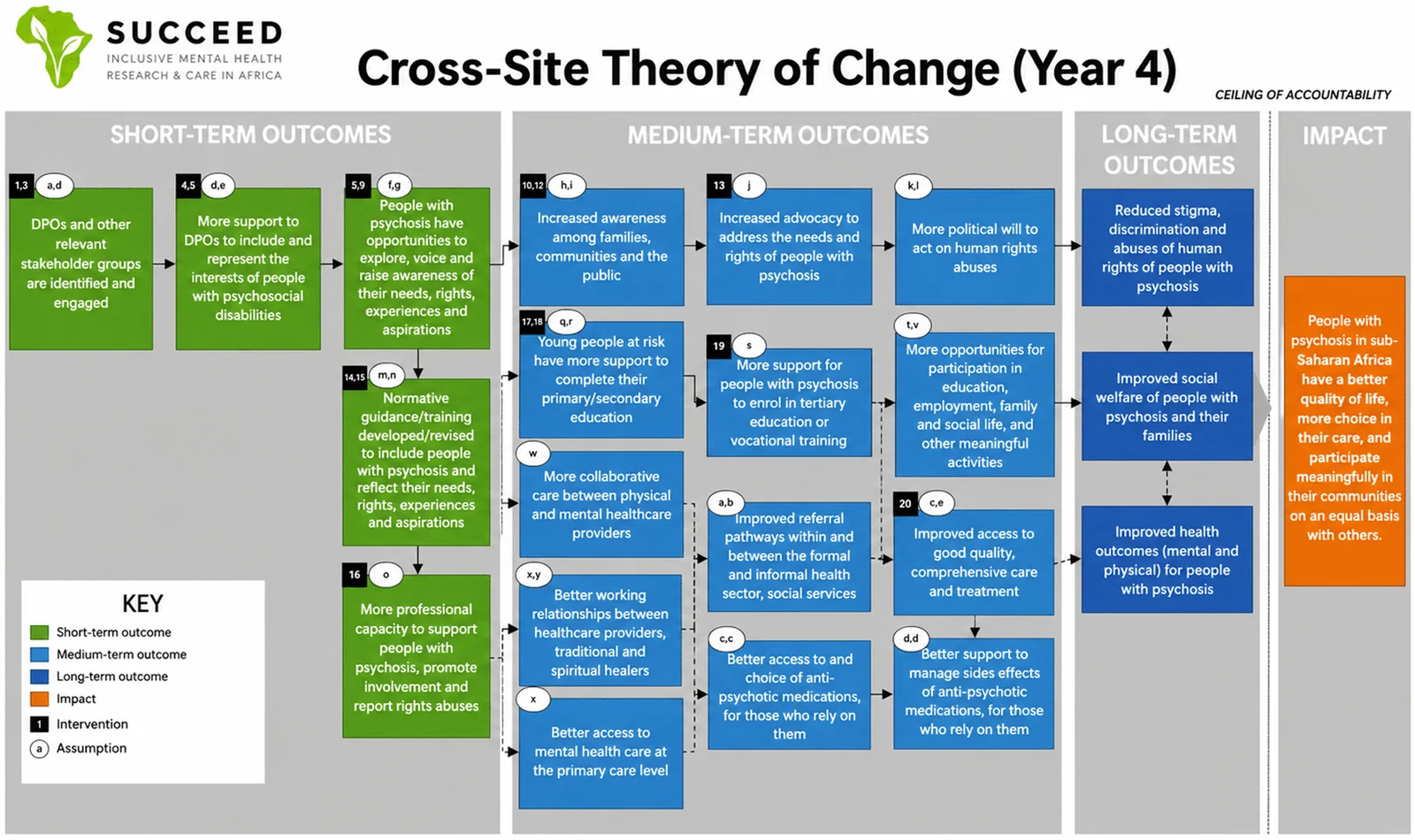
The SUCCEED cross-site Theory of Change Map after piloting.

These changes were incorporated into an updated ToC (Fig 4), and Implementation Manual. A working draft of the SUCCEED Intervention Manual used for the process evaluation and trials is available upon request, though will be reviewed with results from the final research prior to being finalised and published for wider use. In addition, we have completed a description of the intervention used in the SUCCEED trial and process evaluations using the TIDieR framework [18]. See S1 Text.

**Fig 5:**
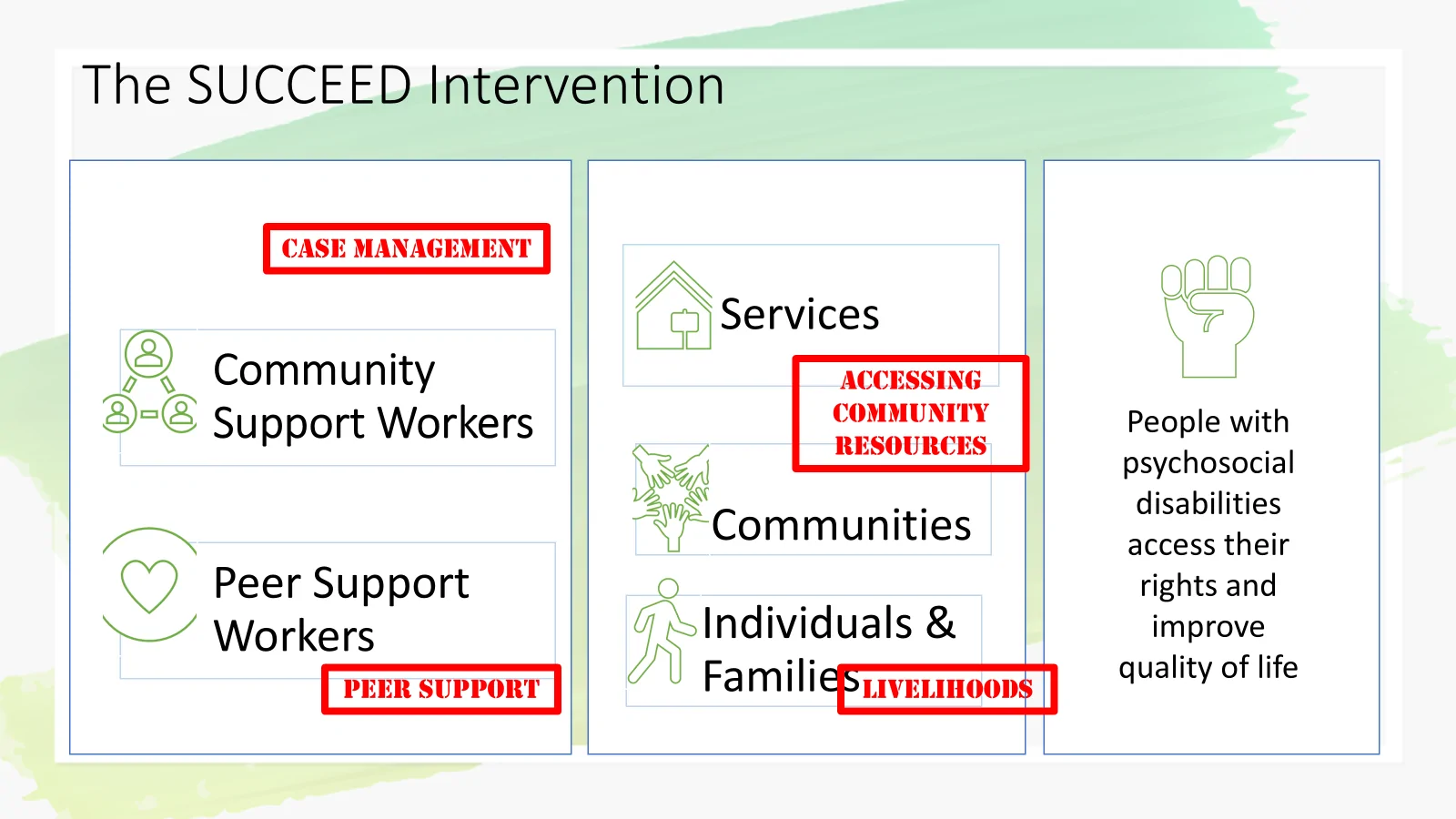
Simplified SUCCEED Theory of Change.

In addition to the finalised ToC and Implementation Manual for use in the full Randomised Control Trials and process evaluations, a summary, accessible version was developed for communication and training purposes (Fig 5).

## Discussion

The process followed in this intervention development is based on a structured, Theory of Change-driven approach that has emerged across a number of similar programmes over several decades [38–40]. The SUCCEED programme was particularly explicit in its efforts to incorporate perspectives of people with lived experience, reflecting the commitment shown to this principle in many global guidelines and frameworks [27,41]. These experiences and practical guidance are available in a co-produced guide with the Global Mental Health Peer Network (now called aves Mental Health) [42].

Participatory processes are rich and can lead to outcomes that differ from those designed by traditionally defined experts alone [22]. Teams described attitude change and new insights from the experience, particularly of working alongside peer researchers and PSWs, who became integral members of teams. The process can also be slow, due to the need for extensive consultation across multiple sites.

SUCCEED sits within a developing tradition of Theory of Change-driven, co-produced intervention development in global mental health. Our methodological approach shares important features with the PRIME programme in five low- and middle-income countries [28], the RISE trial and the Ethiopia CBR development work for schizophrenia [39], and the Mental Health Scale Up Nigeria (mhSUN) programme [40]. In common with UPSIDES [24], we have placed sustained peer support at the centre of the intervention. In common with the mental health Cultural Adaptation and Contextualisation for Implementation (mhCACI) procedure [43], we have treated the fit between intervention and context as an explicit object of design rather than a residual concern. Other work has attempted to fit ToC across very diverse programmes [44], What is distinctive about SUCCEED is the explicit synthesis of four country-specific co-produced ToCs into a single cross-site ToC, while preserving site-level flexibility.

Co-production on this scale carries real costs as well as benefits. Participatory methods across four countries required substantial time and coordination resource, and decisions had to be made about when consensus was achievable and when divergence should be preserved. The emotional labour borne by peer workers and peer researchers, already noted in the literature on peer working, was visible throughout [45]. Equalising voice in mixed-status workshops was difficult even with explicit facilitation measures, and we cannot claim to have fully resolved the asymmetries of language, academic credential and professional authority that shape such spaces. Finally, COVID-19 constrained in-person engagement for almost two years, and although digital platforms enabled continued collaboration, they also limited the informal relational time that co-production often relies on. These experiences suggest that future co-production in multi-country global mental health programmes should budget more explicitly for relationship-building and managing emotional labour, and plan for hybrid in-person and virtual engagement from the outset.

### Limitations

There were some limitations to our approach. ToC as a method for documenting postulated drivers of change is necessarily biased by the opinions and perspectives of the people involved (even in the way that literature is interpreted and applied). This is inevitable in a multifaceted complex intervention, though the fact that ToC also enables testing of assumptions enables later assessment of their accuracy and possibility of addressing these biases.

Precise description of a specific intervention model is necessary for fidelity and any meaningful evaluation [46]. Across four diverse implementation sites, there were inevitable differences in the way that even a co-developed and well understood intervention is implemented. In reviewing these differences, and deciding on commonly agreed changes to an intervention, there is a balance to be struck between consistency/fidelity across sites, and context-dependent adaptation. To an extent the diversity of the intervention offered to the service users in SUCCEED, as a person-centred case management approach, where assessed needs defined a bespoke recovery plan, means some diversity in implementation is built into the model. The GUIDED framework (Item 12) requires reporting of important uncertainties at the end of the intervention development process. This level of flexibility vs fidelity might be framed as uncertainty, but is a core feature of replicating intervention models in different contexts [43]. There were several areas that remained unresolved. How much to provide financial assistance to participants, for example for travel or to pay for medication was extensively debated, based on tensions between moral considerations, and sustainability. The context of COVID-19 both complicated delivery, and stimulated creative solutions for programme management, and potential intervention adaptation.

Similarly, some operational decisions will always need to be made to address unexpected challenges and changes in circumstance, for example during piloting, teams had to make adaptations in real-time delivery, as political or other environmental factors changed. In evaluation of complex interventions, this is a recognised issue, and reasons for changes to initial programme plans transparently documented [46].

A further limitation concerns the representativeness of stakeholder participation in the co-production process. Participants were identified through the networks of research teams and partner civil society organisations, which are likely to have favoured people already connected to services, advocacy, or organised civil society. Despite the measures described above to equalise voice, professional and academic contributors likely remained more influential in some discussions than people with lived experience. Service users from the most marginalised settings, including those with untreated psychosis in remote rural areas, those in traditional institutions, and those experiencing homelessness, are likely to be under-represented. Gender balance among peer support workers and workshop participants varied across sites. These limitations shape both the content of the intervention and the generalisability of its underlying ToC, and argue for continued, deliberate efforts to reach more marginalised voices in subsequent phases of implementation and evaluation.

While there were some adjustments made to the intervention (and training materials), these were not substantial, even after the pilot phase. This indicates that ToC, using a participatory approach with relevant stakeholders, and use of existing evidence, provides a solid foundation for subsequent refinement. Flexibility in implementation is key to efficacy, and can be built into an intervention, but clearly documenting and monitoring the delivery of key active ingredients is essential if replication of proven interventions is to be a meaningful endeavour.

The SUCCEED intervention is not a single treatment but a bundle of mutually reinforcing components, and ToC involves making hypothesised mechanisms of change explicit. We proposed that case management by a Community Support Worker would reduce support gaps by identifying, navigating and linking service users and families to otherwise fragmented community, health and social resources. Peer support can facilitate recovery, reduce internalised stigma, and opening pathways to re-engagement with family and community life, though evidence is mixed and based on weak research methods [47], though more work is being done in this area. Community Mental Health Forums contribute through shifts in community attitudes, active case identification and mapping of informal community supports [33]. Livelihoods activities contribute through restored social roles as active community members, as well as improved income security [48]. Taken together, these components are expected to operate across the domains of CBR, reflecting the social model of disability, addressing both impairment-related needs and the environmental and social barriers to participation [16]. Later quantitative and qualitative results of the SUCCEED study will elucidate the extent to which these hypotheses are confirmed.

### Contextual enablers and barriers

A number of contextual factors influenced the ability of this intervention, and the ToC that informed it, to deliver the intended outcomes. Key issues identified during the pilot related to creating a supportive environment for personnel, clarity of the intervention with a more precise manual, and a focus on practice-based learning. Other enablers included using civil society organisations with strong links to affected communities, and flexibility afforded by digital platforms to maintain cross-site collaboration during COVID-19 restrictions. The sustained involvement of peer researchers and PSWs shaped intervention design and delivery from within the teams, and the governance structures (the Lived Experience Advisory Panel and Local Advisory Groups) created accountable channels for lived experience input. Important barriers included the weakness of formal mental health services and limited statutory social protection to cushion livelihoods, limiting the impact of service-linkage activities; persistent community and household stigma, particularly around psychosis; and the financial vulnerability of participants and of PSWs themselves. Substantial contextual differences across the four countries in language, economic conditions, and civil society capacity required continual balancing between cross-site consistency and local adaptation. These enablers and barriers, and the assumptions that connect them to outcomes, are reflected in the updated ToC and in S3 Table.

### Implications

Methodologically, this paper contributes a multi-country example of how Theory of Change can be used iteratively to develop a complex intervention across diverse settings. We offer a concrete approach for synthesising four site-specific ToCs into a cross-site ToC while preserving site-level flexibility, which may be useful for future multi-country global mental health programmes. Our experience also suggests that co-production at this scale requires explicit investment in relational work, peer supervision structures, and hybrid engagement modalities.

For practice, SUCCEED offers a feasible blueprint for a lay-delivered, community-based intervention for people living with psychosis in low-resource sub-Saharan African settings. The Community Support Worker and Peer Support Worker dyad, complemented by Community Mental Health Forums, peer groups, livelihoods support and structured supervision, is deliberately designed to be adaptable within a common core, and should be transferable to other settings with similarly weak formal systems, active civil society and informal care.

For policy, the intervention aligns with the WHO Community-Based Rehabilitation Guidelines [25], the WHO Mental Health Action Plan [38], the WHO QualityRights framework [27], and the United Nations Convention on the Rights of Persons with Disabilities. It offers a practical illustration of how these frameworks can be operationalised in low-resource African settings, and may support national and sub-national planning for community mental health services.

For research, the paper suggests several priorities: linking ToC with testing of the proposed mechanisms of change through robust evaluation methods; strengthening scalability and sustainability beyond research funding; further work on the wellbeing, retention and career pathways of peer support workers; and continued attention to reaching more marginalised service users in co-production work.

## Supporting information

S1 Table Timeline of the SUCCEED Africa Intervention development. (DOCX)

S2 Table SUCCEED reporting against GUIDED framework. (PDF)

S1 Text TIDieR Checklist for intervention description. (DOCX)

S3 Table Modifications made to the assumptions, components of the intervention and outcomes during the development of the Theory of Change for the evaluation of the SUCCEED trial. (DOCX)
