# Supplementary material for: Co-production using Theory of Change in the development of the SUCCEED Africa community-based rehabilitation intervention for people living with psychosis in West and Southeast Africa
Source: PLOS Ment Health. 2026 Sep 11;3(9):e0000668. doi: 10.1371/journal.pmen.0000668 (PMC13567762; doi:10.1371/journal.pmen.0000668)
Supplement: Supplementary file 2 [file pmen.0000668.s001.docx]

**Supplementary Figure S4.**

**Timeline of the SUCCEED Africa intervention development process, 2019 to 2026.**

| **Year / Period** | **Phase** | **Key activities** |
| --- | --- | --- |
| **2019** | **Programme establishment** | Establishment of the SUCCEED consortium across five sites. Development of guiding principles and values across the country teams, including commitments to co-production, the social model of disability, and sustainability and scalability. Formation of country teams and coordinating centre. |
| **2020 to 2021** | **Situation analysis and formative research** | Four-country situation analyses adapted from WHO QualityRights and PRIME frameworks, covering policy, service infrastructure, financing and local data systems. Formative qualitative research with service users, caregivers, service providers, and traditional and faith healers. Systematic reviews of care for people with psychosis in Africa and of community-based rehabilitation as a service model. Development of site-specific Theory of Change (ToC Stage 1) maps, conducted largely virtually due to COVID-19 travel restrictions. |
| **2022** | **Cross-site intervention design (ToC Stage 2)** | Synthesis of the four site-specific ToC maps into a common cross-site ToC through a series of virtual workshops and a first in-person cross-site meeting in Ibadan, Nigeria. Manualisation of the intervention and development of training materials for Community Support Workers, Peer Support Workers, Supervisors and Peer Supervisors. Agreement on core intervention components: case management, peer support, community engagement through Community Mental Health Forums, and livelihoods support. |
| **2023** | **Pilot training and implementation** | Two-week structured training of CSWs, PSWs and Supervisors in each country, followed by field-based mentorship. Four-month pilot implementation across all four sites, with one CSW and PSW dyad per country supporting approximately ten service users each. Concurrent process evaluation using ENACT competency assessment and structured feedback from providers, service users and caregivers. |
| **Early 2024** | **Post-pilot revision (ToC Stage 3)** | In-person cross-site meeting in Blantyre, Malawi, bringing together country teams, peer researchers and the coordinating centre to review pilot findings. Revision of the cross-site ToC to incorporate learning on peer worker wellbeing, safeguarding, monitoring intensity, livelihoods flexibility, and field-based learning for new staff. Updating of the Intervention Manual and training materials for the full trial. |
| **2024 to 2026** | **Full evaluation** | Full randomised controlled trial implementation and process evaluation across the four sites. Ongoing monitoring of intervention fidelity, service user outcomes, provider experience, and sustainability considerations for post-trial continuation. |
