# Supplementary material for: Co-production using Theory of Change in the development of the SUCCEED Africa community-based rehabilitation intervention for people living with psychosis in West and Southeast Africa
Source: PLOS Ment Health. 2026 Sep 11;3(9):e0000668. doi: 10.1371/journal.pmen.0000668 (PMC13567762; doi:10.1371/journal.pmen.0000668)
Supplement: Supplementary file 3 [file pmen.0000668.s003.docx]

**Supplementary material S3:**

**TIDieR Checklist for intervention description**

1 **Brief name: Provide the name or a phrase that describes the intervention.**

SUpport, Comprehensive Care and EmpowErment for people with psychosocial Disabilities (SUCCEED): A co-produced, rights-based, community intervention delivering peer support, case management, and livelihood support to improve quality of life and social inclusion for people living with psychosis.

2 **Why: Describe any rationale, theory, or goal of the elements essential to the intervention.**

The SUCCEED intervention is grounded in the WHO Community-Based Rehabilitation (CBR) Matrix and a rights-based approach, recognising that the exclusion experienced by people living with psychosis arises not only from health needs but also from social, economic, and rights-related barriers. The intervention was co-produced with people with lived experience of psychosis, families, and community stakeholders to ensure relevance, acceptability, and responsiveness to local contexts.

The intervention is explicitly Theory-of-Change (ToC) driven. The ToC articulates the causal pathways through which peer support, case management, and livelihood-focused activities are expected to improve quality of life and social inclusion, including by strengthening individual agency, mobilising family and community resources, reducing stigma and discrimination, and improving access to services and opportunities. The ToC is treated as a living framework, refined through formative research, piloting, and ongoing learning.

The active components of the intervention are the integrated delivery of peer support by people with lived experience, community-based case management, and livelihoods and empowerment activities aligned to the CBR Matrix. Several also draw on evidence-based frameworks, for example Asset-Based Community Development (ABCD)^[1]^ and Individual Placement and Support^[2]^ for livelihoods. These components are essential to achieving the intended outcomes; omission of any would undermine the intervention’s capacity to address social exclusion, livelihood deprivation, and rights violations that contribute to poor outcomes for people living with psychosis. In this, the intervention is deliberately providing a cross-sectoral and person-centred approach to comprehensive support, rather than a health intervention alone.

3 **What (material): Describe any physical or informational materials used in the intervention, including those provided to participants or used in intervention delivery or in training of intervention providers.**

The SUCCEED intervention uses a defined set of SUCCEED-specific physical and informational materials that guide training, delivery, monitoring, and evaluation of the intervention. Core materials include the **SUCCEED Intervention Manual (field version),** which provides detailed guidance on peer support, case management, livelihoods activities, supervision, and safeguarding procedures across research sites.

Rights-based materials underpinning delivery include tools adapted from **WHO QualityRights**^[3]^, supporting a focus on autonomy, participation, dignity, and non-discrimination. Delivery of peer support draws on **manualised tools and approaches**, including Tree of Life, Circles of Safety (self-disclosure), shared decision-making resources, and recovery planning tools.

Intervention providers are trained using a **structured training curriculum** developed for SUCCEED through UPSIDES^[4]^, RISE^[5]^ trial and mhGAP^[6]^ and refined following pilot testing, combining classroom materials, practical exercises, and field-based coaching resources. Ongoing delivery and implementation quality are supported by standardised **monitoring and evaluation (M&E) materials,** including session records, attendance logs, supervision and fidelity monitoring tools, and routine documentation forms.

While the WHO CBR Matrix informs the overall framework of the intervention, the materials used were contextually adapted and considered SUCCEED-specific resources developed, adapted, and refined to operationalise the intervention in line with its Theory of Change and rights-based design.

4 **What (procedures): Describe each of the procedures, activities, and/or processes used in the intervention, including any enabling or support activities.**

The SUCCEED intervention comprises individual, family, and community level support delivered by Peer Support Workers (PSWs) and Community Support Workers (CSWs). Following identification and screening, service users carry out a needs assessment and identify their primary goals for improved quality of life. They then receive **individual peer support** through regular home visits and one-to-one meetings focused on recovery-oriented support, shared decision-making, coping strategies, and goal setting. In parallel, participants are invited to take part in **group-based peer support** sessions, typically delivered through self-help groups facilitated by PSWs, which provide mutual support, collective problem-solving, and a platform for empowerment and social connection.

Livelihoods activities are delivered primarily through these self-help groups using an Asset-Based Community Development (ABCD) approach, enabling participants and family members to identify existing skills, resources, and opportunities within their communities and to engage in collective or individual income-generating and empowerment activities.

**Case management** is led by CSWs and involves systematic needs assessment, resource mapping, care planning, and coordinated support across health, social, and community services. This includes practical assistance with accessing services, advocacy, accompaniment to appointments where needed, and referral to appropriate health or social care providers.

**Community Mental Health Forums** are organised to raise awareness, reduce stigma, and engage families, leaders, and service providers. These forums also function as points for community sensitisation, programme visibility, and ongoing dialogue around psychosis and inclusion to reduce myths and stigma.

Intervention delivery is supported by **continuous supervision and peer mentoring**, with regular supervisory meetings, peer-to-peer support structures, and fidelity monitoring to sustain quality, safeguard participants and providers, and support adaptive problem-solving during implementation.

5 **Who provided: For each category of intervention provider (for example, psychologist, nursing assistant), describe their expertise, background and any specific training given.**

The SUCCEED intervention is delivered by **Peer Support Workers (PSWs)** and **Community Support Workers (CSWs)**, who are specifically recruited and trained for this role. It may be in another version of the intervention that existing health or social care staff could play the role of CSW, but PSWs will always need to be identified and specifically trained as rarely exist in established services. PSWs are people with lived experience of psychosis who are “on the road to recovery”, with sufficient literacy, numeracy and fluency in local languages to complete training and deliver structured peer support. CSWs are community-based workers (typically recruited through partner civil society organisations) with at least three years of secondary education, strong local knowledge, and the literacy and numeracy required for case management, documentation, and participation in supervision. Both PSWs and CSWs complete a structured multi-week training programme, combining classroom teaching and field-based coaching, and their competence is assessed using standardised tools before and during intervention delivery, with ongoing supervision and mentoring. Supervisors within partner organisations oversee PSWs and CSWs, provide regular supervision sessions, and support fidelity and safeguarding; clinicians may receive referrals but **do not** deliver the intervention itself. A specific Peer Supervisor also provides a supportive role for PSWs and is available to provide cover should PSWs need time off or a break.

6 **How (mode of delivery; individual or group): Describe the modes of delivery (such as face to face or by some other mechanism, such as internet or telephone) of the intervention and whether it was provided individually or in a group.**

The SUCCEED intervention is delivered **face-to-face** using a combination of **individual- and group-based modalities**, tailored to participants’ needs and contexts. **Individual delivery** occurs primarily through one-to-one home visits conducted by Peer Support Workers and Community Support Workers, focusing on recovery-oriented peer support, case management, and practical problem-solving. Intervention providers may also **accompany participants to health or social services** to facilitate access, advocacy, and engagement with care.

**Group-based delivery** takes place through structured self-help groups for people living with psychosis and, separately, for family members, typically involving small groups facilitated by PSWs or CSWs.

In addition, **Community Mental Health Forums** are delivered at the community level and involve larger groups of community members, leaders, and service providers, with an emphasis on awareness-raising, stigma reduction, and community engagement. These take place at the start of the programme to engage with communities, gain buy-in for the programme, challenge stigma, and facilitate case identification. They are then repeated annually.

All contacts are **interactive** and initiated either by the intervention providers as part of scheduled activities or collaboratively with participants in line with a person-centred approach. Delivery occurs across **home, community, and service-linked settings**, as appropriate for different elements of the intervention, and depending on preference of service user and in agreement with the provider.

Remote contact using standard mobile phones for ‘checking in’ occurs between physical meetings based on the judgement of the PSW and CSW. There is otherwise no routine use of remote or digital modalities, though information management for monitoring, and remote supervision would be possible in future implementation.

7 **Where: Describe the type(s) of location(s) where the intervention occurred, including any necessary infrastructure or relevant features.**

The SUCCEED intervention is delivered across multiple locations, reflecting its community-based and person-centred design. A substantial proportion of the intervention takes place in participants’ homes, where Peer Support Workers and Community Support Workers conduct individual peer support, case management activities, and follow-up visits. Home-based delivery is central to reaching participants who may have limited mobility, face stigma, or have reduced access to facility-based services.

Additional delivery occurs in community venues such as community centres, faith-based or educational spaces, and other locally accessible locations used for group self-help sessions and livelihoods activities. Community Mental Health Forums are held in larger public community settings to enable broad participation by community members, leaders, families, and service providers, and to support awareness-raising and stigma reduction. Examples include open spaces near markets or village/town centres, or town halls.

While the intervention itself is not delivered within clinical services, it is closely linked to health-facility contexts through referral and accompaniment, with intervention providers supporting participants to engage with appropriate health or social care services as needed. This combination of home-, community-, and service-linked settings is integral to the feasibility, acceptability, and scalability of the SUCCEED intervention.

8 **When and how much: Describe the number of times the intervention was delivered and over what period of time including the number of sessions, their schedule, and their duration, intensity or dose.**

The SUCCEED research programme delivered the intervention over a **12-month implementation period**, however, it is designed to be a sustained, rolling programme, where service users would be recruited, supported, and discharged based on need. Following identification and screening, eligible participants receive an **initial home visit** for needs assessment, and subsequently engage in defining their specific care package to address their needs and goals, and being supported in achieving this.

The **intended “dose”** of the intervention is defined by **regular, contact**, a minimum of once a month, combined with flexibility in frequency and intensity according to participant needs and preferences. As part of a person-centred and recovery-oriented approach, the **intensity of contact is participant-directed** rather than fixed, with intervention providers and participants jointly determining the timing, focus, and frequency of individual visits, group participation, and case management activities.

In addition to these monthly contacts, facilitation of other support like peer support groups and livelihood activities, work placement, health appointments, etc take place.

Intervention engagement is expected to be **continuous over time**, rather than confined to a short or discrete set of sessions, and may vary across participants and as levels of need change. The actual amount of intervention received therefore differs between participants.

9 **Tailoring: In tailored interventions, not all participants receive an identical intervention. Was this intervention planned to be personalised, titrated or adapted?**

The SUCCEED intervention is intentionally tailored, with the content, frequency, and intensity of activities guided by participant needs, preferences, and circumstances rather than delivered as a fixed package. Tailoring is participant-led and person-centred, recognising the diversity of experiences, priorities, and levels of support required by people living with psychosis.

Individual-level tailoring occurs following initial and ongoing **needs assessment** conducted by Peer Support Workers and Community Support Workers, which informs decisions about the type of support provided (with an emphasis on peer support, case management, and livelihoods activities, as well as family psychoeducation and promoting appropriate use of medical services), the balance between individual and group engagement, and the frequency of contact. Intervention intensity may be increased or reduced over time in response to changes in participants’ wellbeing, goals, social context, or engagement.

Tailoring decisions are supported by structured guidance within the **SUCCEED Intervention Manual**. Adaptations at both individual and intervention levels are documented through routine monitoring and supervision processes, as well as regular meetings of CSWs and PSWs, enabling reflection, learning, and consistency in application while preserving flexibility to respond to participants’ evolving needs.

10 **Modification: Unforeseen modifications to the intervention can occur during the course of the study, particularly in early studies. Was this intervention modified during the course of the study?**

Study-level modifications to the SUCCEED intervention were made as part of a deliberate **learning and refinement process**, particularly following formative research and pilot implementation. The **Theory of Change** underpinning the intervention was treated as a **living framework** and updated iteratively to incorporate empirical learning from qualitative research, pilot findings, and early implementation experience. These updates informed refinements to assumptions, pathways, and emphasis across intervention components, while preserving the core elements of peer support, case management, and livelihoods activities.

**Post-pilot refinements** were undertaken to improve feasibility, acceptability, and clarity of intervention delivery across sites. These modifications included adjustments to training content, operational guidance, and delivery processes to better reflect local contexts and implementation realities. All modifications were **learning-driven rather than ad hoc**, occurred prior to or during early phases of full implementation, and were documented through consortium processes and updated intervention materials. The refined intervention therefore differs from the initial pilot version in operational detail and clarity, but not in its underlying rationale, core components, or intended outcomes.

Examples of modifications included:

- training becoming more practical (eg with more role play teaching), and being extended to include supervised field experience
- reducing the intensity of documentation in monitoring so that supervisors spent more time providing practical support to CSWs and PSWs;
- more flexibility being given to PSWs to take time off if needed (without risking income or their jobs) and more support provided by the Peer Supervisor
- addition of ‘floating PSWs’ who were able to cover for PSWs who were unable to work
- more precise definition of how livelihood activities were managed in groups, so there was more consistency, while preserving flexibility for individual preferences to be the guide to activities

11 **How well (planned): Fidelity refers to the degree to which an intervention happened in the way the investigators intended it to.**

Intervention fidelity in the SUCCEED study refers to the extent to which the intervention was delivered and received in accordance with the intended design across sites. Given the complexity of the intervention, fidelity was addressed at multiple levels, including **training of providers**, **delivery of intervention components**, and **participant engagement**.

Fidelity was maintained and assessed using **pre-specified strategies and tools**. These included standardised **attendance logs** to capture participation in individual visits, group sessions, and community activities; routine **monitoring and evaluation (M&E) forms** completed by Peer Support Workers and Community Support Workers to document delivery, content, and frequency of activities; and **structured supervision and mentoring sessions** in which delivery challenges, deviations, and adaptations were reviewed. Supervisors played a central role in supporting adherence to core intervention components and documenting acceptable adaptations.

In addition, fidelity was examined as part of the **Theory-of-Change-driven process evaluation**, aligned with the **Medical Research Council (MRC) Process Evaluation Framework**, which assessed implementation fidelity, coverage, and uptake using mixed methods, including routine monitoring data, observations, and qualitative inquiry. Together, these approaches enabled systematic assessment of how closely the delivered intervention aligned with the intended model, and provided essential context for interpretation of study outcomes.

12 **How well (actual): For various reasons, an intervention, or parts of it, might not be delivered as intended, thus affecting the fidelity of the intervention.**

There were several topics around which levels of flexibility were needed, based on being responsive to users’ individual needs and preferences. In relation to management of peer support groups, in some cases, family members were part of the meetings (mainly to participate in livelihood activities), but guidance was shared to ensure that at least some protected time without families in attendance was included.

**References**

1. Haines A. Asset-based community development. In: An introduction to community development 2014 Nov 26 (pp. 67-78). Routledge.
2. Bond GR, Drake RE, Becker DR. An update on individual placement and support. World Psychiatry. 2020 Sep 15;19(3):390.
3. Funk M, Bold ND. WHO’s QualityRights initiative: Transforming services and promoting rights in mental health. Health and human rights. 2020 Jun;22(1):69.
4. Goldfarb Y, Grayzman A, Meir LG, Grundman SH, Rabinian M, Lachman M, Epstein PG, Ben-Dor IA, Naaman A, Puschner B, Moran GS. UPSIDES mental health peer support in face of the COVID-19 pandemic: Actions and insights. Community Mental Health Journal. 2024 Jan;60(1):5-13.
5. Asher L, Birhane R, Weiss HA, Medhin G, Selamu M, Patel V, De Silva M, Hanlon C, Fekadu A. Community-based rehabilitation intervention for people with schizophrenia in Ethiopia (RISE): results of a 12-month cluster-randomised controlled trial. The Lancet Global Health. 2022 Apr 1;10(4):e530-42.
6. Keynejad RC, Dua T, Barbui C, Thornicroft G. WHO Mental Health Gap Action Programme (mhGAP) Intervention Guide: a systematic review of evidence from low and middle-income countries. BMJ Ment Health. 2018 Feb 1;21(1):30-4.
