# Supplementary material for: Co-production using Theory of Change in the development of the SUCCEED Africa community-based rehabilitation intervention for people living with psychosis in West and Southeast Africa
Source: PLOS Ment Health. 2026 Sep 11;3(9):e0000668. doi: 10.1371/journal.pmen.0000668 (PMC13567762; doi:10.1371/journal.pmen.0000668)
Supplement: Supplementary file 4 [file pmen.0000668.s004.docx]

**Supplementary information S2:**

**Modifications made to the assumptions, components of the intervention and outcomes during the development of the theory of change for the evaluation of the SUCCEED trial**

| **1.0 Peer Support and User’s voice** | |
| --- | --- |
| **1.1 Assumptions** | **Modifications/revisions** |
| DPOs and other key stakeholders are willing and able to participate. | Local Organisations for Persons with Disabilities (OPDs), Non Governmental organisations (NGOs) and other key stakeholders are willing and able to participate |
| Resources are sufficient to support accessible and effective meetings and communications | OPDs and NGOs can work with their available resources and participate effectively in meetings |
| Virtual meetings and communication can provide an equally accessible and effective alternative to face-to-face meetings, where required (e.g., due to Covid-19 restrictions) | Virtual meetings and communication can provide an effective alternative to face-to-face meetings, where required (e.g., due to Covid-19 restrictions), though accessibility needs to be addressed. |
| DPOs do not face operational challenges/threats (e.g., lack of funding) that inhibit their functioning | OPDs can overcome operational challenges and threats such as lack of funding that inhibit their functioning. |
| There are no barriers to people with psychosocial disability participating in DPOs | People with psychosocial disabilities and their families are willing and able to participate in OPDs, NGOs, and self-help groups. |
| People with psychosocial disabilities and their families are willing and able to participate in self-help groups | Deleted |
| People with psychosocial disabilities can safely self-disclose/participate in awareness-raising, despite stigma and discrimination, including from families. | Despite stigma and discrimination people with psychosocial disabilities and their families are willing to self-disclose and participate in awareness-raising, despite stigma and discrimination. |
| **1.2 Interventions** | **Modifications/Revisions** |
| Carry out regular LAGs, LEAPs, ToC workshops, CAG & other stakeholder meetings | Carry out regular stakeholder meetings such as Local Advisory Groups, Lived Experience Advisory Panels, to promote regular stakeholder engagement. |
| Carry out sensitization activities targeted to DPOs | Carry out sensitization activities |
| Assist in identifying people with psychosocial disabilities to join DPOs, as needed. | Work with local OPDs and NGOs to ensure participation of people with psychosocial disabilities. |
| Establish self-help groups for people with psychosocial disabilities and their families, linked to DPOs | Establish self-help groups for people with psychosocial disabilities and their families. |
| Co-produce & co-deliver training on UN CRPD & rights of people with psychosocial disabilities (based on QualityRights) to self-help groups | Co-produce and co-deliver a training incorporating human rights approach to self-help groups (eg based on QualityRights) |
| **2.0 Awareness and access to rights** | |
| **2.1 Assumptions** | **Modifications/revisions** |
| Key stakeholders will be willing and able to translate advocacy training into action, without expecting further incentives | Key stakeholders will be willing and able to translate advocacy training into action, without expecting unreasonable incentives |
| **3.0 EDUCATION AND LIVELIHOODS** | |
| **3.1 Assumptions** | **Modifications/revisions** |
| Appropriate opportunities for tertiary education/vocational training exist in the area. | Appropriate opportunities for tertiary education and vocational training exist in the area and are available for people with psychosocial disabilities. |
| SUCCEED team have or can partner with other organisations that have the skills/expertise and resources to establish livelihoods activities | Other organisations that have the skill, expertise and resources to establish livelihoods activities can be identified and linked with for referral. |
| **3.2 Interventions** | **Modifications** |
| Involve school authorities in advocating/making provisions for flexible study opportunities to accommodate unique needs of people with psychosocial disabilities | Engage with school authorities to advocate for provision for flexible study opportunities to accommodate unique needs of people with psychosocial disabilities. |
| Establish livelihoods activities/skills development for out-of-school youth | Establish livelihoods activities and skill development for out-of-school youth (with psychosocial disabilities) |
| **4.0 Health Care** | |
| **4.1 Assumptions** | **Modifications** |
| Barriers to access do not interrupt participants’ uptake of referrals | Services are willing and able to handle possible increases in demand through referrals |
| Relationships between services do not deteriorate | Removed |
| It is possible to make drugs available (especially second-line drugs) at affordable prices | Support is provided to make drugs available where service users cannot access or afford them |
| It is possible to effectively manage side effects, with the resources available (human resources, drugs, special accommodations, e.g. at work/home) | Health services are able to provide good quality health care, including managing side-effects well. |
| Factors beyond our control do not negatively impact quality (e.g., hospital strikes) | Additional support and solutions can be provided for service users when events beyond the programme’s control occur (eg health worker strikes) |
| **4.2 Interventions** | **Modifications** |
| Establish e-health/telemedicine options and SMS reminders for services, as appropriate | Establish e-health and telemedicine options and SMS reminders for services, as appropriate and if quality of e-health option service is adequate and acceptable to users |

* Note the change in preferred terminology from DPO (Disabled Persons’ Organisation) to OPD (Organisation of Persons with Disabilities), which occurred during the timeframe of the programme.
