# Supplementary material for: Co-production using Theory of Change in the development of the SUCCEED Africa community-based rehabilitation intervention for people living with psychosis in West and Southeast Africa
Source: PLOS Ment Health. 2026 Sep 11;3(9):e0000668. doi: 10.1371/journal.pmen.0000668 (PMC13567762; doi:10.1371/journal.pmen.0000668)
Supplement: Supplementary file 5 [file pmen.0000668.s006.docx]

**Response to Reviewers**

*PLOS Mental Health*

*Manuscript: Co-production using Theory of Change in the development of the SUCCEED Africa community-based rehabilitation intervention for people living with psychosis in West and Southeast Africa*

We thank the Editor and three Reviewers for their careful engagement with our manuscript and for comments that have considerably improved the clarity, rigour and reach of the paper. We have revised the manuscript in line with each comment, and all additions are shown in red in the revised manuscript. Our responses below indicate how each comment has been addressed and the approximate location of the change.

**Journal Requirements**

| **No.** | **Reviewer Comment** | **Author Response** | **Page / Line of clean document** |
| --- | --- | --- | --- |
| 1 | Please amend your detailed Financial Disclosure statement in full sentences containing the exact wording you wish to be published, clarifying all sources of funding, initials of authors receiving each grant, and whether any authors received a salary from funders. | Proposed Financial Disclosure statement to read: ‘The SUCCEED Africa programme was funded by UK Aid from the UK Foreign, Commonwealth and Development Office (FCDO) through the Research Programmes Consortiums grant (GB-EDU-133903-301134). All authors received salary support from this grant during the development of the intervention described in this paper. The funders had no role in study design, data collection and analysis, decision to publish, or preparation of the manuscript.’ | Cover letter / Financial Disclosure |
| 2 | Please provide separate figure files in .tif or .eps format. | All five figures (Figure 1: CBR Framework; Figure 2: Nigeria in-country ToC; Figure 3: Cross-site ToC pre-pilot; Figure 4: Cross-site ToC post-pilot; Figure 5: Simplified SUCCEED ToC) will be re-exported at 300 dpi or higher and submitted as separate .tif files meeting PLOS figure requirements. The low-resolution ToC diagram raised by Reviewer 1 (R1.11) will be replaced with a high-resolution version. | All figures |

**Editor Comments**

| **No.** | **Reviewer Comment** | **Author Response** | **Page / Line** |
| --- | --- | --- | --- |
| E1 | Please consider review of the Figures, particularly Figure 2, to consider if they can be more simple or clear. | We have revised the figures to make them clearer (alongside using the appropriate format. However, the ToC figures are the result of the collective decisions described (and hence are results) and cannot be edited in relation to their detail. | Figures 1, 2, 3, 4, 5 |
| E2 | Please consider whether there are contextual factors, enablers and barriers that might influence the Theory of Change ability to deliver outcomes. Could these be elaborated in the text and figures? | A new subsection has been added suggesting contextual factors, enablers and barriers. In addition the Supplementary Material S2 lists assumptions and how they were perceived to have influenced the implementation success. | Page 23; S2 |
| E3 | Please consider whether your data and theories of change could include attention to proposed mechanisms, which can be instructive for practitioners. | A new sub-section has been added to the Discussion making explicit the hypothesised mechanisms. In summary: (i) case management by a CSW reduces treatment gaps by linking service users to fragmented community and formal resources; (ii) PSWs contribute through role-modelling of recovery, reduction of internalised stigma, and experiential credibility; (iii) Community Mental Health Forums shift normative community attitudes and enable active case identification; (iv) livelihoods activities contribute to recovery through restored social roles, income and self-efficacy; and (v) the combination operates on the social model of disability by addressing both impairment-related and environmental barriers. These mechanisms are now also flagged in the narrative accompanying the cross-site ToC. | Page 22 (lines 595-611) |
| E4 | Please review your Discussion. The so what of this paper feels insufficiently developed. Please consider how your findings and methods align with work by others. | The Discussion has been expanded to situate our methods and findings more explicitly within the existing literature. We now compare our ToC-driven co-production approach with other programmes in LMICs, including CBR development work in psychosis, and programmes with similar intervention components. | Page 20 (lines 518-543) |
| E5 | Please develop an implications section, expanding on how this study contributes to the wider literature and knowledge, both with respect to methods and the end outputs. | A new "Implications" sub-section has been added at the end of the Discussion, | Page 23 (new sub-section line 628-653) |

**Reviewer 1**

| **No.** | **Reviewer Comment** | **Author Response** | **Page / Line** |
| --- | --- | --- | --- |
| R1.0 | General comment: This manuscript addresses an important topic and presents a valuable participatory approach to developing a community-based rehabilitation intervention for people living with psychosis in African settings. | We thank the Reviewer for this positive overall assessment and for the detailed and constructive comments that follow. Each has been addressed below. | N/A |
| R1.1 | The manuscript does not clearly articulate specific objectives at the end of the introduction. | A new paragraph has been added at the end of the Introduction setting out four specific objectives:  (i) to describe the iterative, co-produced ToC process used to develop the SUCCEED intervention across four sub-Saharan African settings; (ii) to present the evolving ToC maps and resulting intervention components; (iii) to describe the final intervention using the GUIDED and TIDieR frameworks; and (iv) to reflect on methodological strengths, limitations, enablers and barriers of this participatory development approach. | Page 4 (line 110-117) |
| R1.2 | The manuscript lacks sufficient methodological detail on the co-production process, including stakeholder recruitment, representation of service users and caregivers, and handling of power dynamics in workshops. | A new subsection has been added to the Methods ("Co-production processes and stakeholder engagement") describing: (i) which stakeholder groups were involved at each stage (people with lived experience of psychosis, family caregivers, peer researchers, PSWs, CSWs, supervisors, mental health professionals, community leaders, civil society organisations and researchers); (ii) how participants were identified and invited (through existing relationships of site teams and partner civil society organisations, with targeted outreach to ensure representation of people with lived experience and caregivers at each workshop); (iii) the composition of the LEAP and LAGs; and (iv) measures taken to manage power dynamics, including separate lived-experience-only reflection spaces, use of peer facilitators, structuring workshops so professional contributions were responded to by lived experience contributions, and explicit framing of all contributions as equally valuable. | Page 7 (lines 179-184 and 203-230) |
| R1.3 | The manuscript does not clearly explain how workshop data were synthesised, how the final ToC was derived, and how disagreements were resolved. | A new paragraph has been added to the Development Process describing how workshop outputs were synthesised. In summary: workshop notes, flipchart outputs and photographs of ToC maps were compiled by site focal persons; a core writing group produced draft cross-site ToCs by mapping shared outcomes, mechanisms and assumptions across sites and identifying divergences; each draft was circulated to all sites for feedback; divergences were resolved through structured virtual and face-to-face cross-site meetings (ultimately including annual in-person meetings in Nigeria, Malawi and Zimbabwe) with a consensus-seeking approach. Where consensus could not be reached, the default was to retain site-level flexibility rather than impose a cross-site position. The LEAP was consulted on all major versions of the ToC. | Page 11 (lines 311-323) |
| R1.4 | The manuscript does not clearly establish how the ToC informed the design of the SUCCEED intervention components. | We have added explicit linking text in the Results section indicating how each intervention component derives from the cross-site ToC: (i) case management emerged from ToC assumptions about fragmented services needing active navigation; (ii) peer support emerged from outcomes relating to hope, self-efficacy and reduced internalised stigma; (iii) livelihoods activities emerged from outcomes relating to restored social roles and reduced household economic strain; and (iv) community engagement through CMHFs emerged from outcomes on stigma reduction and identification of unmet need. | Results, Intervention Components.  Page 14 (lines 388-395) |
| R1.5 | The manuscript provides insufficient detail on the intervention, including who delivers it, training requirements, and expected duration and intensity. | Additional detail has been added to the Intervention Delivery subsection and Table 1. Reference is made to the full TIDieR description in Supplementary Material S3. | Page 14 (line 398-414)  Table 1; S3 |
| R1.6 | The manuscript does not compare the co-production approach with other intervention development models in global mental health and offers limited critical reflection on challenges encountered. | The Discussion has been expanded to compare our approach with PRIME, RISE and the Ethiopia CBR development work, mhSUN, UPSIDES, and the mhCACI framework. A new paragraph also provides critical reflection on challenges encountered during co-production, including: time and cost implications of participatory methods across four countries; emotional labour on peer workers; the difficulty of equalising voice in mixed-status workshops; the tension between site-level flexibility and cross-site fidelity; and the practical constraints imposed by COVID-19 on in-person deliberation. | Page 20 (line 521-546) |
| R1.7 | The manuscript provides only partial explanation of contextual similarities and differences across settings, and implications for generalisability. | A new paragraph has been added to the Methods (Setting) and referenced in the Discussion summarising contextual similarities and differences with reference to full details in the situation analysis paper.  However, more detail is included in the separate SUCCEED Situation analysis paper, which is referenced. | Page 5 (lines 125-138) |
| R1.8 | The manuscript does not acknowledge possible selection bias in stakeholder participation, dominance of professional voices, and limited representation of service users from rural or marginalised communities. | A new limitations paragraph has been added to the Discussion explicitly acknowledging: (i) that participants in co-production workshops were identified through networks of research teams and partner civil society organisations, which may have favoured those already engaged with services or advocacy; (ii) that despite efforts to equalise voice, professional and academic contributors likely remained dominant in some discussions; (iii) that service users from the most marginalised settings (including those living with untreated psychosis in rural areas, those in traditional institutions, and those experiencing homelessness) are likely to be under-represented; and (iv) that gender balance among peer support workers and workshop participants varied across sites. We discuss implications for the generalisability of the intervention model. | Page 22 (line 528-588) |
| R1.9 | The manuscript does not describe in detail ethical procedures related to the co-production workshops, including ethical approval bodies, consent procedures, and protections for potentially vulnerable participants. | A new subsection ("Ethics and safeguarding") has been added to the Methods. The overall SUCCEED programme received ethical approval from the London School of Hygiene & Tropical Medicine Research Ethics Committee and from each country partner institution (University of Ibadan / UI-UCH Ethics Committee for Nigeria; Kamuzu University of Health Sciences Research Ethics Committee for Malawi; University of Makeni and the Sierra Leone Ethics and Scientific Review Committee for Sierra Leone; and the Medical Research Council of Zimbabwe for Zimbabwe). | Page 12  (new subsection; p329-347) |
| R1.10 | The manuscript uses terms interchangeably that may imply different approaches, for example co-production, co-design, and participatory intervention development. | We have harmonised terminology throughout. "Co-production" is now used consistently as the overarching term and defined explicitly in the Methods with reference to Epstein and more recent conceptualisations in global mental health. "Co-design" is used only where specifically referring to the design workshops; "participatory intervention development" is no longer used interchangeably. A short clarifying sentence has been added in the Methods to make this terminological choice explicit. | Sub-section, page 7 |
| R1.11 | The manuscript includes a Theory of Change diagram with low resolution. | The ToC diagrams (Figures 2, 3 and 4) should be redrawn and submitted as separate high-resolution files (minimum 300 dpi, .tif format) in compliance with PLOS figure requirements. Figure 2 should be simplified per Editor comment E1. | All figures |

**Reviewer 2**

| **No.** | **Reviewer Comment** | **Author Response** | **Page / Line** |
| --- | --- | --- | --- |
| R2.1 | Lines 58 to 59: The language used may unintentionally reinforce stigma by listing prisons and street homelessness as likely outcomes. Please reframe to emphasise systemic gaps rather than individual trajectories. | We thank the Reviewer for this important point. The sentence has been reframed to foreground the systemic failure of care pathways rather than individual trajectories. The original sentence has been revised to:  ‘In the absence of accessible, appropriate care pathways, these systemic gaps can leave individuals without adequate support, exposing them to informal or traditional treatments that may provide little more than containment, or risk prison or street homelessness.’ | Page 2 (lines 52-57) |
| R2.2 | Line 70: the term "key worker" is unclear and needs elaboration. | A definitional clarification has been added in the relevant sentence:  ‘A key worker, or case manager, is a single designated worker who takes responsibility for coordinating a service user's care and support across health, social and livelihood sectors.’ We have also added a reference to ground this definition in literature.  This is consistent with our later use of Community Support Worker. | Page 3 (lines 70-73) |
| R2.3 | Line 92: "care providers in the community" needs to be defined. | A clarifying sentence has been added:  ‘In this paper, care providers in the community refers to the range of formal and informal actors who provide some form of care or support to people with mental health conditions in everyday settings, including family and household caregivers, community and religious leaders, traditional and faith healers, community health workers, peer supporters, and civil society organisations.’ | Page 4 (page 96-100) |
| R2.4 | Line 325: The phrasing may inadvertently place responsibility on PSWs for being unable to continue. Please reframe to highlight structural and workplace factors. | Thank you for pointing this out. This is certainly not our intention, and the paragraph has been reframed to locate the challenge in the structural working conditions within which PSWs operated rather than in individual capacity. The revised text reads:  ‘The structural conditions within which peer support workers operated, including role ambiguity, limited locally-relevant training and supervision infrastructure, financial precarity, and broader systemic pressures within under-resourced mental health services, created challenging working conditions that at times made it difficult for PSWs to sustain their roles.’ | Page 17 (page 452-456) |
| R2.5 | General comment: the paper could be strengthened by incorporating or foregrounding lived experience narratives or reflections from participants. | We thank you for pointing out this valuable way of reflecting lived experience in our paper. We are committed to such visibility of lived experience voices, but while the authorship of this paper includes people with lived experience (in the role of peer researchers), we feel that adding this element to this paper at this late stage it might risk tokenism. There are dedicated papers focusing on lived experience perspectives in SUCCEED in preparation, and participants in the Peer Support Worker role are included in the pilot evaluation. | N/A |

**Reviewer 3**

| **No.** | **Reviewer Comment** | **Author Response** | **Page / Line** |
| --- | --- | --- | --- |
| R3.0 | General comment: I enjoyed reviewing this manuscript. I appreciate the efforts to keep it grounded within the realities of people's everyday lives. | We thank the Reviewer for the generous comments and for the specific suggestions below, all of which have been addressed. |  |
| R3.1 | Page 3, last paragraph: consider mentioning Nigeria first when discussing innovations in community-based mental health care, rather than starting with a Global North example. | We have restructured the paragraph to lead with African examples of innovation in community-based mental health care. Thomas Adeoye Lambo's Aro village system in Nigeria is now presented as the first example, followed by other African and Global South examples, with Geel, Belgium cited as one of a number of older international examples. | Page 3 (lines 60-67) |
| R3.2 | Page 4, reference 7: the scoping review cited focuses largely on non-African contexts, which might be worth mentioning. | We have added a qualifying phrase noting that this evidence base is drawn predominantly from high-income and non-African settings:  ‘Most case management literature is drawn from high-income and non-African settings, but more recent work from sub-Saharan Africa, like the RISE trial of community-based rehabilitation for schizophrenia in Ethiopia [14], is beginning to build a more contextually relevant evidence base.’ | Page 3 (line 74-78) |
| R3.3 | Page 4, line 77: capitalise Global South or explain the choice not to do so. | Global South and Global North have been capitalised consistently throughout the manuscript, in line with current convention in global health and postcolonial scholarship. | Throughout |
| R3.4 | Page 5, paragraph 1: you said the study is co-produced. Co-produced with whom? | Clarified in the Methods section on co-production. | Page 8 (line 203-211) |
| R3.5 | Page 6, Target population: clarify whether participants require a clinical diagnosis. | We have clarified that SUCCEED does not require a prior formal clinical diagnosis. Eligibility is based on community-level identification of presentations consistent with psychosis (including schizophrenia-spectrum and bipolar affective disorder) using the Community Informed Detection Tool, followed by a structured screening process operable by non-specialists. This reflects our deliberate design choice to enable identification in settings where formal diagnostic services are inaccessible. Specialist confirmation is sought where accessible but is not a precondition for engagement with the intervention. This clarification has been added in the Target Population section. | Page 6 (line 148-156) |
| R3.6 | Methods section: could benefit from more theoretical backing, particularly on co-production and social model of disability. | Additional theoretical grounding has been added. For co-production, we now cite Epstein, and Jakobsson and colleagues' scoping review of co-production in psychosis research, and the SUCCEED and Global Mental Health Peer Network basic guide. For the social model of disability, we have added references to Oliver's foundational work, the UN Convention on the Rights of Persons with Disabilities, Mulvany's analysis of its application to mental disorder, and WHO CBR Guidelines. A short paragraph has been added locating SUCCEED at the intersection of these two traditions. | Page 7 (lines 179-184) and page 9 (lines 235-239) |
| R3.7 | Page 9, first paragraph: realist or realistic? Two different meanings. | Corrected. The intended meaning is "realist" (in the technical sense of realist evaluation methodology associated with Pawson and Tilley). The sentence has been corrected and a brief definition added: "realist process evaluation, which examines the mechanisms through which interventions produce outcomes in particular contexts." More detail will be given in the relevant publications at a later date. | Page 9 (lines 254-258) |
| R3.8 | Page 9, paragraph 2: please clarify why you are drawing on QualityRights and PRIME. | A sentence has been added explaining the rationale:  ‘WHO QualityRights was selected because it provides a structured, rights-based framework for assessing mental health services and identifying gaps against internationally recognised standards, directly relevant to our situation analysis. PRIME was selected because it offers a tested template for situation analyses specifically in LMIC mental health system contexts, including practical tools for mapping policy, financing, workforce and service infrastructure. Together they allowed us to combine a rights-based lens with a systems-mapping approach appropriate to our four settings.’ | Page 10 (lines 285-290) |
| R3.9 | Page 15, paragraph 1: please clarify "recruitment was carried out independent of research team" and list other eligibility criteria for CSWs and PSWs. | Clarified: "Recruitment of CSWs and PSWs was led by the partner civil society organisation in each country (ASIDO Foundation in Nigeria, MeHUCA in Malawi, the Mental Health Coalition in Sierra Leone, and ZIMNAMH in Zimbabwe) rather than directly by the academic research team, in order to model the recruitment pathway envisaged for any future scale-up beyond a research context." Both roles required signed commitment to the SUCCEED safeguarding and confidentiality standards. | Page 17 (lines 431-438) |
| R3.10 | Page 16, point 2 on safeguarding: please provide more detail. | The safeguarding point has been expanded in two places; as part of a dedicated ethics and safeguarding section, and as an outline of how this was strengthened following piloting. | Page 12 (lines 340-348) and Page 18 (lines 463-471 |
| R3.11 | Please provide an outline of the project timeline. | A project timeline section has been added and as a new Supplementary Figure S4 | Page 10 (lines 264-275) and S4 |

.
