# Supplementary material for: Co-production using Theory of Change in the development of the SUCCEED Africa community-based rehabilitation intervention for people living with psychosis in West and Southeast Africa
Source: PLOS Ment Health. 2026 Sep 11;3(9):e0000668. doi: 10.1371/journal.pmen.0000668 (PMC13567762; doi:10.1371/journal.pmen.0000668)
Supplement: Peer review history

# Peer review: Decision Letter 0

**DOI:** 10.1371/journal.pmen.0000668.r001

**Authors:** Kaaren Mathias

19 Mar 2026

PMEN-D-26-00020

Co-production using Theory of Change in the development of the SUCCEED Africa community-based rehabilitation intervention for people living with psychosis in West and Southeast Africa

PLOS Mental Health

Dear Dr. Eaton,

Thank you for submitting your manuscript to PLOS Mental Health. After careful consideration, we feel that it has merit but does not fully meet PLOS Mental Health’s publication criteria as it currently stands. Therefore, we invite you to submit a revised version of the manuscript that addresses the points raised during the review process.

Please submit your revised manuscript by May 03 2026 11:59PM. If you will need more time than this to complete your revisions, please reply to this message or contact the journal office at mentalhealth@plos.org. When you're ready to submit your revision, log on to https://www.editorialmanager.com/pmen/ and select the 'Submissions Needing Revision' folder to locate your manuscript file.

Please include the following items when submitting your revised manuscript:

- A letter that responds to each point raised by the editor and reviewer(s). You should upload this letter as a separate file labeled 'Response to Reviewers'.
- A marked-up copy of your manuscript that highlights changes made to the original version. You should upload this as a separate file labeled 'Revised Manuscript with Track Changes'.
- An unmarked version of your revised paper without tracked changes. You should upload this as a separate file labeled 'Manuscript'.

Guidelines for resubmitting your figure files are available below the reviewer comments at the end of this letter.

We look forward to receiving your revised manuscript.

Kind regards,

Kaaren R Mathias, PhD

Academic Editor

PLOS Mental Health

Journal Requirements:

1. Please amend your detailed Financial Disclosure statement. This is published with the article. It must therefore be completed in full sentences and contain the exact wording you wish to be published.

a. Please clarify all sources of funding (financial or material support) for your study. List the grants (with grant number) or organizations (with url) that supported your study, including funding received from your institution.

b. State the initials, alongside each funding source, of each author to receive each grant.

c. If any authors received a salary from any of your funders, please state which authors and which funders.

2. Please provide separate figure files in .tif or .eps format.

For more information about figure files please see our guidelines:  LINK

https://journals.plos.org/mentalhealth/s/figures

https://journals.plos.org/mentalhealth/s/figures#loc-file-requirements

If the reviewer comments include a recommendation to cite specific previously published works, please review and evaluate these publications to determine whether they are relevant and should be cited. There is no requirement to cite these works unless the editor has indicated otherwise.

Additional Editor Comments (if provided):

Thank you for this paper submission with interesting and important methods and findings.

Please address the comments from reviewers. From an Editorial perspective:

Please consider review of the Figures - particularly Figure 2 to consider if they can be more simple or clear.

Please consider whether there are contextual factors / enablers and barriers that might influence the TOC ability to deliver outcomes – could these be elaborated in the text and figures if you have data that would support this.

Please also consider whether your data and your theories of change could include some attention to proposed mechanisms – these can be very instructive to provide insight to practitioners.

Please review your Discussion – the ‘so what’ of this paper feel insufficiently developed -please consider how your findings and methods align with work by others researching or publishing in this area.

I would also like to see further development of an ‘implications’ section – please expand how does this study contribute to the wider literature and knowledge – both with respect to methods and the end outputs?

Thank you,

[Note: HTML markup is below. Please do not edit.]

Reviewers' comments:

Reviewer's Responses to Questions

**Comments to the Author**

1. Does this manuscript meet PLOS Mental Health’s publication criteria?

Reviewer #1: Yes

Reviewer #2: Yes

Reviewer #3: Yes

**********

2. Has the statistical analysis been performed appropriately and rigorously?

Reviewer #1: N/A

Reviewer #2: Yes

Reviewer #3: N/A

**********

3. Have the authors made all data underlying the findings in their manuscript fully available (please refer to the Data Availability Statement at the start of the manuscript PDF file)?

The PLOS Data policy

Reviewer #1: Yes

Reviewer #2: Yes

Reviewer #3: Yes

**********

4. Is the manuscript presented in an intelligible fashion and written in standard English?

Reviewer #1: Yes

Reviewer #2: Yes

Reviewer #3: Yes

**********

Reviewer #1: This manuscript addresses an important topic and presents a valuable participatory approach to developing a community-based rehabilitation intervention for people living with psychosis in African settings.

However, the manuscript currently:

1. Does not clearly articulate specific objectives at the end of the introduction.

2. Lacks sufficient methodological detail on the co-production process i.e., stakeholder recruitment procedures, representation of service users and caregivers, and handling of power dynamics in workshops. Because co-production is the core methodology, insufficient detail limits reproducibility.

3. Does not clearly explain how workshop data were synthesized, how the final ToC was derived, and how disagreements were resolved. This weakens the analytic rigor of the intervention development process.

4. Does not clearly establish how the ToC informed the design of the SUCCEED intervention components. This appears to be a conceptual gap rather than merely a presentation issue.

5. Provides insufficient detail on the intervention, including who delivers it (e.g., community health workers or peer supporters), the training requirements, and the expected duration and intensity.

6. Does not compare the co-production approach used with other intervention development models in global mental health and offers limited critical reflection on the challenges encountered during co-production.

7. Provides only a partial explanation of the contextual similarities and differences across the study settings, and the implications of these differences for generalizability, given that the intervention spans multiple African contexts.

8. Does not acknowledge possible selection bias in stakeholder participation, dominance of professional voices in workshops, and limited representation of service users from rural or marginalized communities.

9. Does not describe in detail ethical procedures related to the co-production workshops i.e., ethical approval bodies, consent procedures for participants, and protections for potentially vulnerable participants (e.g., people living with psychosis).

10. Uses some terms interchangeably but may imply different approaches e.g., ‘co-production’, ‘co-design’, and ‘participatory intervention development’.

11. Includes a ToC diagram with low resolution.

Reviewer #2: Line 58-59 - The language used in this sentence may unintentionally reinforce stigma toward people with lived experience of mental health challenges. By listing prisons and street homelessness as likely outcomes, the phrasing risks implying a connection between mental health service users and criminality. While the authors are aiming to highlight the consequences of inadequate service provision, it may be helpful to reframe this sentence in a way that emphasizes systemic gaps in care and support rather than potential individual trajectories that could be perceived as stigmatizing. For example, the authors could focus more explicitly on the lack of accessible, appropriate care pathways and how these systemic failures can leave individuals without adequate support.

Line 70 – “through the support of a key worker” – It is unclear as to what a key worker is. Further elaborate for clarity.

Line 92 – “from care providers in the community” – it would be of value to describe what is defined as a care provider in the community.

Line 325 - The phrasing in this sentence may inadvertently place responsibility on peer support workers (PSWs) themselves for being unable to continue in their roles. Given that PSWs often work within structurally constrained environments such as under-resourced services, unclear role definitions, limited supervision, or insufficient organizational support, it may be more appropriate to frame this issue in relation to workplace conditions rather than individual capacity. Revising the language to highlight the structural factors that contribute to stress could strengthen the analysis and avoid inadvertently suggesting that PSWs themselves are the source of the problem. For instance, the authors might consider discussing how role ambiguity, lack of localised, culturally relevant training support, or broader systemic pressures within mental health services can create challenging working conditions for PSWs. This reframing would acknowledge the value of peer support work while situating the challenges within the systems in which PSWs operate.

General comments: The paper could potentially be strengthened by incorporating or foregrounding lived experience narratives or reflections drawn from participants involved in the SUCCEED project. Including brief narrative examples, quotations, or reflections from peer support workers, peer researchers, or other participants with lived experience could add depth to the analysis and illustrate how the themes discussed in the manuscript manifest in practice. Even a small number of illustrative narratives or examples could help provide richer contextualization of the findings and highlight the experiential dimensions identified within the SUCCEED project.

Thank you to the authors for this important and valuable work. Overall, I commend the authors for your work and for contributing to a vital and evolving area of research and practice.

Reviewer #3: Overall, I enjoyed reviewing this manuscript. I appreciate the efforts to keep it grounded within the realities of people's everyday lives, both in terms of what may be meaningful to them as well as what may be realistic in regard to longer term sustainability of the intervention. It also reads very nicely.

Please find below a few comments from my end:

- p. 3, last paragraph. You mention innovations in mental heath community-based care. Why not mention Nigeria first, especially given the geographical focus of the paper? I know this might be a small comment, but I think it's important we not always look at the Global North as always the first examples of innovation.

- p.4, ref 7. That scoping review focuses largely on non-African context - might be worth mentioning

- p. 4, line 77. Capitalise Global South or explain choice not to do so

- p.5, paragraph 1. You said that the study is co-produced. Co-produced with whom?

- P.6, Target population. Can you clarify whether that is people with clinical diagnosis? Or not necessarily, given the flexible approach to sample definition?

- Methods section. I think the whole paragraph could benefit from a bit more theoretical backing, particularly sections on co-production and social model of disability. Especially because, as you say, 'co-production' has taken on so many different meanings and applications. Seeing a few more references would be good to better situate your understanding and approach to it.

- p.9, first paragraph. Realist or realistic? Two different meanings

- p. 9, paragraph 2. Please clarify why you are drawing on QualityRights and PRIME.

- p.15, paragraph 1. Can you please clarify what you mean by recruitment was carried independent of research team? Also where there other eligibility criteria in place for recruitment, other than being a CSW or PSW?

-p.16, point 2 on safeguarding. Can you provide a bit more detail around that, and how it was put in place?

-As a whole, you might want to provide an outline of the timeline of the project?

**********

what does this mean?). If published, this will include your full peer review and any attached files.

**Do you want your identity to be public for this peer review?**  If you choose “no”, your identity will remain anonymous but your review may still be made public.

For information about this choice, including consent withdrawal, please see our Privacy Policy

Reviewer #1: No

Reviewer #2: No

Reviewer #3: **Yes:** Alma Ionescu

**********

[NOTE: If reviewer comments were submitted as an attachment file, they will be attached to this email and accessible via the submission site. Please log into your account, locate the manuscript record, and check for the action link "View Attachments". If this link does not appear, there are no attachment files.]

# Author comment: Author response to Decision Letter 1

**DOI:** 10.1371/journal.pmen.0000668.r002

6 May 2026

Attachment Submitted filename: Response_to_Reviewers_SUCCEED_PLoSMH May26.docx

# Peer review: Decision Letter 1

**DOI:** 10.1371/journal.pmen.0000668.r003

**Authors:** Kaaren Mathias

9 Jul 2026

Co-production using Theory of Change in the development of the SUCCEED Africa community-based rehabilitation intervention for people living with psychosis in West and Southeast Africa

PMEN-D-26-00020R1

Dear Mrs Greenley,

We are pleased to inform you that your manuscript 'Co-production using Theory of Change in the development of the SUCCEED Africa community-based rehabilitation intervention for people living with psychosis in West and Southeast Africa' has been provisionally accepted for publication in PLOS Mental Health.

Before your manuscript can be formally accepted you will need to complete some formatting changes, which you will receive in a follow up email. A member of our team will be in touch with a set of requests.

Please note that your manuscript will not be scheduled for publication until you have made the required changes, so a swift response is appreciated.

IMPORTANT: The editorial review process is now complete. PLOS will only permit corrections to spelling, formatting or significant scientific errors from this point onwards. Requests for major changes, or any which affect the scientific understanding of your work, will cause delays to the publication date of your manuscript.

If your institution or institutions have a press office, please notify them about your upcoming paper to help maximize its impact. If they'll be preparing press materials, please inform our press team as soon as possible -- no later than 48 hours after receiving the formal acceptance. Your manuscript will remain under strict press embargo until 2 pm Eastern Time on the date of publication. For more information, please contact mentalhealth@plos.org.

Thank you again for supporting Open Access publishing; we are looking forward to publishing your work in PLOS Mental Health.

Best regards,

Kaaren R Mathias, PhD

Academic Editor

PLOS Mental Health

***********************************************************

Thank you for all the work and responses to reviewers and the updates which have strengthened this paper. Please can you give an update on the availability of workshop outputs, ToC maps, facilitator notes and summary reports generated by the study. We are now happy to accept this paper for publication. Please note that there are a couple of comments and very minor reviewer comments that need to be addressed before we can proceed to production of this paper.

thanks,

Kaaren Mathias

Reviewer Comments (if any, and for reference):

Reviewer's Responses to Questions

**Comments to the Author**

Reviewer #1: All comments have been addressed

Reviewer #2: All comments have been addressed

Reviewer #3: All comments have been addressed

**********

publication criteria?

Reviewer #1: Yes

Reviewer #2: Yes

Reviewer #3: Yes

**********

3. Has the statistical analysis been performed appropriately and rigorously?

Reviewer #1: Yes

Reviewer #2: Yes

Reviewer #3: N/A

**********

4. Have the authors made all data underlying the findings in their manuscript fully available (please refer to the Data Availability Statement at the start of the manuscript PDF file)?

The PLOS Data policy

Reviewer #1: Yes

Reviewer #2: Yes

Reviewer #3: Yes

**********

5. Is the manuscript presented in an intelligible fashion and written in standard English?

Reviewer #1: Yes

Reviewer #2: Yes

Reviewer #3: Yes

**********

Reviewer #1: 1. Minor editorial issues remain e.g., a typo "co-deign" instead of "co-design" (line 204 and 215).

2. Authors claim that they have presented all data relevant to this article in the manuscript. The Editor may ask for clarification regarding availability of workshop outputs, ToC maps, facilitator notes and summary reports generated by the study.

Reviewer #2: The paper shows a clear improvement in structure and readability, with a noticeably better flow and overall coherence throughout. I commend the authors for their thorough and constructive engagement with the reviewer comments, which has clearly strengthened the manuscript.

My only remaining comment is that the references should be carefully checked for accuracy and completeness. For example, Reference 42 appears to be missing or cannot be located. Thank you for your clear commitment and dedication in the field.

Reviewer #3: I am satisfied that the authors have responded to my comments and those of fellow reviewers. The level of detail, particularly methodological, in the updated manuscript has created a meaningful change.

Just one small comment: Line 215, the word 'co-design' is misspelled.

**********

what does this mean?). If published, this will include your full peer review and any attached files.

**Do you want your identity to be public for this peer review?**  If you choose “no”, your identity will remain anonymous but your review may still be made public.

For information about this choice, including consent withdrawal, please see our Privacy Policy

Reviewer #1: No

Reviewer #2: No

Reviewer #3: No

**********
